# Mode-Selective Nonlinearity in a Monolithic Slitted Piezoelectric Energy Harvester: Symmetry-Controlled Internal Resonance, Saturation, and Differential Bistability

**DOI:** 10.3390/s26206416

**Published:** 2026-10-11

**Authors:** Saad F. Almokmesh, Bashar B. Alzuwayer

**Affiliations:** 1 Department of Mechanical Power and Refrigeration Technology, College of Technological Studies, Public Authority for Applied Education and Training (PAAET), Kuwait City 70654, Kuwait; 2 Department of Automotive Mechanics and Marine Engineering Technology, College of Technological Studies, Public Authority for Applied Education and Training (PAAET), Kuwait City 70654, Kuwait; bb.alzuwayer@paaet.edu.kw

**Keywords:** piezoelectric energy harvesting, slitted cantilever, internal resonance, saturation, bistability, mode selectivity, symmetry breaking

## Abstract

Nonlinear elements in vibration energy harvesters conventionally act on coordinates that every structural mode shares, so added functionality detunes the power-carrying resonance. We analyse a different placement: a permanent-magnet pair mounted inside the slit of a monolithic slitted piezoelectric cantilever, acting only on the difference in the prong deflections. Because this coordinate is odd under the device mirror symmetry, an exact selectivity theorem holds: at equal tip masses, the element is invisible to the symmetric mode family at every order in amplitude, while a rank-one lever places the antisymmetric resonance across 33–60 Hz. Deliberate mass mistuning *μ* then opens a gated 2:1 internal resonance (coupling asymptotically ∝ *μ*^2^, activation threshold ∝ *μ*^−3^), verified against direct integration of the full cubic model to 0.1–2% and cross-checked by an independent finite element model. The two regimes trade off: at the mistuned design point, the first resonance shifts by 5.9%, and thresholdless transfer syphons 38% of its matched-load power at 0.05 g. The device serves a 43.3 Hz line at 16.6–20.1 μW, where the symmetric platform delivers 0.15–0.21 μW, integrates 448 vs. 354 μW⋅Hz over the band (1.27×), with unloaded/matched activation thresholds 0.017 g/0.079 g and an optimal load fraction *ρ*^⋆^ ≈ 0.34.

## 1. Introduction

Vibration energy harvesting converts the kinetic energy of ambient structural motion into electrical power for wireless sensor nodes and other low-power electronics, a possibility identified early by Williams and Yates [1] and developed into practical piezoelectric generators by Roundy and Wright [2]. The canonical device is a piezoelectric cantilever operating at its fundamental resonance, for which Erturk and Inman established the closed-form distributed-parameter electromechanical theory [3]. Two decades of subsequent work, surveyed in Refs. [4,5], converge on the same central limitation: a linear resonator delivers useful power only in a narrow band around one frequency, while ambient sources drift, contain multiple lines, and vary in amplitude.

The dominant remedy has been to make the harvester nonlinear. Monostable Duffing devices bend the resonance peak [6,7]; bistable piezomagnetoelastic beams exploit inter-well jumps for broadband gains, from the stochastic inverted pendulum of Cottone et al. [8] and the harmonic piezomagnetoelastic beam of Erturk et al. [9,10] through the systematic nonlinear dynamic analyses of Stanton et al. [11] and the review of Pellegrini et al. [12]; rotatable-magnet and multistable variants extend the idea further [13,14,15]. Magnets are likewise the standard tool for resonance-frequency tuning [16,17]. Parametric excitation offers resonant amplification beyond the direct-drive bound, but at the cost of an activation threshold, and Jia and co-workers devoted a design programme to passively lowering it [18,19,20]. Comprehensive treatments of these nonlinear routes are given in Refs. [20,21,22].

A second, structurally distinct route to bandwidth is multimodality: shaping the structure so that several resonances fall usefully close together. L-shaped beam–mass assemblies [23], multiresonant clamped-clamped beams with side branches [24], two-degree-of-freedom bodies [25,26], branched beams [27], trident geometries [28,29], and split cantilevers in which parallel grooves create prong-like branches with individual tip masses [30] all deliver multiple power peaks. The present authors’ contribution to this family is the slitted cantilever: a single monolithic beam, one continuous machined member, with the compact slit-mounted element of this paper as its only attachment, whose outer span is divided by a longitudinal slit into two prongs carrying independent tip masses. Our previous experimental and finite element study demonstrated that slit length, slit width, and tip masses jointly control the spacing and coupling of the first two resonance peaks and their anti-resonance dips [31], and a companion optimisation study established the multi-variable design methodology for the underlying cantilever platform [32]. A closed-form branched-beam theory of the linear slitted device [33] sharpened those findings into two limitations of the linear design, both re-derived in a self-contained manner in Appendix A so that the present argument does not rest on the specific numbers of the companion work. Within the practical mass range, the bare ratio f_2/f_1 is confined to a locus set by slit length, while f_1 is set almost entirely by total tip mass, so the pair can be steered only along that locus. Decisively, the antisymmetric mode so placed cannot be powered; the second power peak remains parasitically weak under base excitation because the antisymmetric family carries near-zero base participation at symmetric masses.

Meanwhile, the nonlinear-dynamics community has shown that internal resonance (IR), the commensurable coupling of two structural modes, is itself a harvesting resource. Chen and Jiang introduced the concept with a snap-through electromagnetic archetype [34] and a piezoelectric counterpart; Cao, Leadenham and Erturk analysed an M-shaped realisation [35]; Xiong, Tang and Mace achieved 2:1 IR by attaching a properly tuned auxiliary oscillator [36], and the same group exploited axially loaded beams with an added oscillator [37]. Yang and Towfighian coupled IR with magnetic nonlinearity for low-frequency harvesting [38]; frequency up-conversion has been achieved magnetically [39] and through internal resonance [40]; Yan, Taha and Tan analysed the closely related autoparametric architecture [41]. The underlying mechanism of quadratic modal coupling at 2:1 commensurability, producing saturation of the driven mode and transfer of the surplus to its partner, has an older and rigorous pedigree in the autoparametric absorber of Haxton and Barr [42], the saturation-based vibration controllers of Oueini, Nayfeh and Pratt [43], and the classical perturbation framework of Nayfeh and Mook [44]. Its value is equally established at the microscale, where internal resonance stabilises the frequency of nonlinear micromechanical oscillators [45,46] and coherent intermodal energy transfer has been observed directly [47]. Recent work has even mapped the transition between internal resonance and mode localisation as coupling strength is modulated [48], phenomena rooted in the eigenvalue-loci veering of disordered structures [49].

Surveying this literature reveals a structural blind spot with one family of near misses that must be named. In cut-out two-beam harvesters, magnets placed between an inner and an outer beam do act on a relative internal coordinate inside a monolithic in-plane structure: the canonical cut-out platform of Wu, Tang, Yang and Soh [50] and its outer–inner magnetic descendant by Tian et al. [51] extended to an experimentally validated tri-stable variant by Li et al. [52] are the closest prior art to the present construction. But the inner and outer beams of that family are not mirror-symmetry partners: no exact selection rule protects either mode family, the primary resonance is not invariant under the coupling, and the coupling strength obeys no symmetry-order-parameter law. In every other internally resonant or nonlinear harvester cited above, the nonlinear element acts on a coordinate that all modes share: a tip magnet working against ground, an auxiliary oscillator attached to the host, geometric nonlinearity of the beam itself, or an axial load. Three consequences follow. First, the nonlinearity that creates the desired modal coupling also detunes and distorts the primary resonance—the very peak that carries most of the harvested power—so linear reliability and nonlinear functionality trade off against each other. Second, because the coupling is not selective, the commensurability condition must be engineered by global structural tuning (beam lengths, added oscillators), which perturbs everything at once. Third, auxiliary oscillators and external magnet frames sacrifice the monolithic compactness that made the cantilever attractive in the first place. To the best of the authors’ knowledge, no harvester has been reported in which the nonlinearity is mode-selective by symmetry in the exact sense of Proposition 1: attached at a location where an entire mode family has exactly zero participation, so that the linear behaviour of that family is preserved not approximately but identically, while the orthogonal family acquires a strong, independently tunable nonlinear channel. Related saturation- and coupling-based routes have since been demonstrated directly: Harne, Sun and Wang leveraged nonlinear saturation in an L-shaped harvester [53], Aouali, Kacem and Bouhaddi functionalized internal resonance in magnetically coupled resonators for wideband hybrid harvesting [54], and Zou et al. showed that a single nonlinear-force element can be customised for harvesting, isolation, and energy-sink duty alike [55]. Symmetry-breaking mode localization of the kind noted above [48,49] has been reported directly in parametrically excited microbeam-resonator pairs [56].

The slitted cantilever is, as we show, the natural host for exactly this construction, and the slit itself is the natural location. This paper introduces a slit element: a compact force pair in the reference implementation, two small permanent magnets facing each other across the slit at the prong tips that transmit force between the prongs and therefore respond only to the difference Δ = *w_a_* − *w_b_* of their deflections. We keep the word monolithic deliberately, and scope it once: unlike the external magnet frames and auxiliary oscillators discussed above, everything here except the two glued magnets and their shims is a single machined beam the element adds ∼0.4 g and no external structure, ground reference, or auxiliary resonator. Because Δ is odd under the mirror symmetry of the slitted geometry, the element decouples exactly from the even (symmetric) mode family whenever the tip masses are equal, and couples to the odd (anti-symmetric) family with full strength. Deliberate mass mistuning then reopens a coupling channel of precisely controlled magnitude. The contributions are as follows:

1. An exact mode-selectivity theorem (Section 2): At equal tip masses, the slit element is absent from the symmetric-mode equations at every order in amplitude, because the differential participation *d_r_* of every symmetric mode vanishes identically together with the root field of every anti-symmetric mode. The base-excited resonance *f*_1_ is therefore linear and pinned. This result is verified numerically to machine precision (|*d_r_*|∼10^−14^, Δ*f*_1_ = 0 across element stiffness sweeps).
2. A rank-one linear lever and closed-form bistability threshold: The element’s linear stiffness *k*_1_ enters the modal problem as a rank-one update acting only on the anti-symmetric block, moving *f*_2_ over tens of hertz (33–60 Hz demonstrated), while *f*_1_ is invariant, completing the two-target inverse design the linear device lacked and producing a supercritical pitchfork to a bistable differential state at $k_{1}^{\mathrm{cr}}=-{\left[\sum_{r\in A}d_{r}^{2}/\omega_{r}^{2}\right]}^{-1}$, with the two-mode estimate accurate to 0.2%.
3. Symmetry-breaking scaling laws for internal resonance: The mistuning law *d*_1_ = *χμd*_2_ (verified to 0.05%), the coupling law *Λ* ∝ *μ*^2^, and the activation law *A*_thr_ ∝ *μ*^−3^ (fitted slopes 0.97, 1.76, −2.62 for *μ* ≤ 0.3, approaching the asymptotic exponents 1,2,−3 as *μ* → 0), which together classify the accessible 2:1 and 1:3 regimes by two independent symmetry breakings: mounting offset and mass mistuning.
4. A verified nonlinear response: Multiple-scales modulation equations, re-derived symbolically, agree with direct integration of the full cubic reduced model to 0.1–2% across the linear branch, the activation threshold, the saturation plateau, and the subharmonic spillover, for a design point with *A*_thr_ = 0.017 g.
5. Two validity laws with design force: The parasitic self-quadratic strength $P=2\zeta_{1}{\left({\tilde{\omega }}_{1}/{\tilde{\omega }}_{2}\right)}^{2}{\left({\tilde{d}}_{2}/{\tilde{d}}_{1}\right)}^{2}$ is independent of element strength, so clean autoparametric operation requires strong mistuning; and the cubic Duffing detuning is annihilated identically by mounting the magnets at the offset $\Delta_{0}^{⋆}≃0.246\;g_{0}$, the zero of the fourth derivative of the dipole interaction law. The mounting offset simultaneously acts as a lean dial for the coupled frequency response (hardening/right-leaning below $\Delta_{0}^{⋆}$, symmetric at it, mildly softening above).

The paper is organised as follows. Section 2 presents the device concept and the nonlinear branched-beam electromechanical model, including the selectivity theorem, the two-port reduced-order model, and the multiple-scales analysis of both excitation cases. Section 3 describes the exact eigensolver, the calibration of the reconstructed geometry, and the verification battery. Section 4 presents and interprets the results of the linear lever, the static bifurcation, the scaling laws, the 2:1 internal resonance, and the offset study, with the physics of each discussed in place. Section 5 condenses the findings into design guidelines, Section 6 states limitations honestly, and Section 7 concludes.

## 2. Device Concept and Nonlinear Electromechanical Model

### 2.1. Configuration

**Figure 1:**
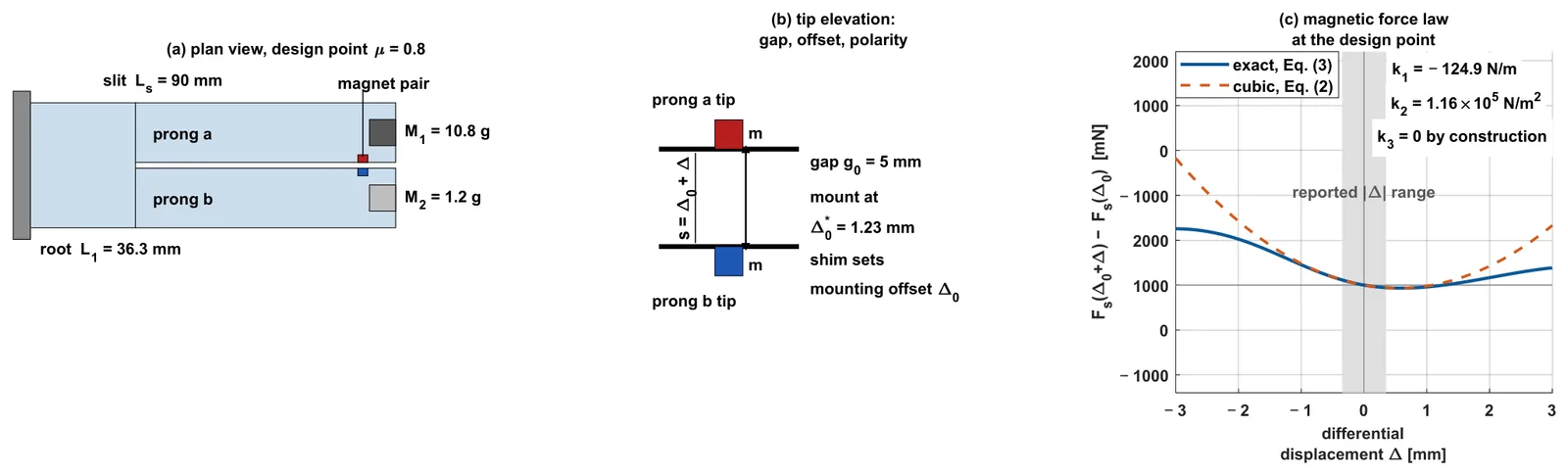
Device schematic. (**a**) Plan view of the slitted harvester at the 2:1 design point: Common root of length *L*_1_, slit of length *L_s_*, prongs with unequal tip masses *M*_1_/*M*_2_ = 10.8/1.2 g (*μ* = 0.8), and the magnet pair mounted across the slit at the prong tips. (**b**) Tip elevation: Gap *g*_0_, shim-set mounting offset Δ_0_, and polarity convention. Parallel moments (repulsive, *C* > 0) give *k*_1_ < 0, lower *f*_2_, and reach bistability beyond *C_cr_*; opposed moments (attractive, *C* < 0) give *k*_1_ > 0 and raise *f*_2_. Mounting at $\Delta_{0}^{⋆}=0.246\;g_{0}$ annihilates the cubic coefficient. Panel (**a**)’s width value and explanatory notes are consolidated here instead: cantilever width *b* = 43.3 mm; PZT-5A (0.36 mm) on aluminium (0.71 mm), base excitation *w_B_*(*t*) at the clamp. The tip-mass blocks and magnets are drawn as separate elements purely for legibility; *M*_1_ and *M*_2_ are each prong’s lumped tip inertia, magnet, and shim included. (**c**) The magnetic force law at the design point (μ = 0.8): the exact dipole force of Equation (3) against the cubic truncation of Equation (2) actually used in the analysis, showing where the truncation remains accurate: within the shaded band of amplitudes reported in this paper (|Δ| ≤ 0.35 mm) the two laws differ by less than 0.4% of the force; the deviation reaches 6% at 1 mm.

Figure 1 shows the device. A cantilever of length *L* and width *b* carries a PZT-5A layer of thickness *h_p_* on an aluminium substrate of thickness *h_s_*. A longitudinal slit of width *w_s_* runs from *x* = *L*_1_ to the free end, dividing the outer span into prongs *a* and *b* of width *b_p_* = (*b* − *w_s_*)/2 with independent tip masses *M*_1_, *M*_2_; the inner segment (the root) retains the full width. All segments share one laminate, so the per-unit-width bending stiffness $\hat{\mathrm{EI}}$ and mass $\hat{m}$ are common. The electrodes are segmented into a root electrode and one electrode per prong; the sum wiring *v*_Σ_ (series-aiding) and the difference wiring *v*_Δ_ (*a* − *b*) realise a two-port harvester, generalising the switchable anti-resonance identified in the linear study [31]. Prong a carries *M*_1_ and prong b carries *M*_2_ throughout.

The single structural addition of this work is the slit element: a force pair mounted at *x_m_* inside the slit, transmitting a transverse force *F_s_* from prong to prong. Its defining kinematic property is that *F_s_* depends on the prong displacements only through their difference measured from a built-in static offset Δ_0_,

Δ(*t*) = *w_a_*(*x_m_*,*t*) − *w_b_*(*x_m_*,*t*),  *F_s_* = *F_s_*(Δ_0_+Δ). (1)

Expanding about the operating point,

*F_s_*(Δ_0_+Δ) − *F_s_*(Δ_0_) = *k*_1_Δ+*k*_2_Δ^2^ + *k*_3_Δ^3^ + 𝒪(Δ^4^), (2)

with *k_n_* set by the physical element. For the reference magnet pair with dipole moments perpendicular to their in-plane separation *g*_0_, the interaction energy is

(3) $$U_{s}\left(s\right)=C\;\frac{g_{0}^{2}-2s^{2}}{{\left(g_{0}^{2}+s^{2}\right)}^{5/2}},\;\;s=\Delta_{0}+\Delta ,$$

where *C* ∝ *μ*_0_*m_a_m_b_* carries the polarity: *C* > 0 (parallel moments, repulsive) gives *k*_1_ = *C* *U_s_*″(Δ_0_) < 0 at small offsets, while *C* < 0 (opposed moments, attractive) gives *k*_1_ > 0. Explicitly, *F_s_*(*s*) = *C* *U_s_*′(*s*) (equivalently *F_s_* = −Π*_s_*′(*s*) for the potential Π*_s_*≡−*C* *U_s_*), which fixes the sign of every *k_n_* unambiguously. The offset Δ_0_ independently controls the quadratic and cubic coefficients *k*_2_ = *C* *U_s_*‴(Δ_0_)/2, $k_{3}=C\;U_{s}^{\left(4\right)}\left(\Delta_{0}\right)/6$. A contact stop or elastomer bridge realises the same expansion with material constants instead.

### 2.2. Modal Projection and the Differential Participation Vector

Each segment obeys an Euler–Bernoulli electromechanical balance of the Erturk–Inman type [3], with the slit force entering the prong equations as ±*F_s_* 𝛿(*x* − *x_m_*). Projecting on the mass-normalised modes $\varphi_{r}=\left(\varphi_{r}^{\left(1\right)},\varphi_{r}^{a},\varphi_{r}^{b}\right)$ of the *linear branched* structure (the exactly solvable model of the linear study) gives the reduced equations

(4) $${\ddot{q}}_{r}+2\zeta_{r}\omega_{r}{\dot{q}}_{r}+\omega_{r}^{2}q_{r}+d_{r}\;F_{s}\;\left(\Delta_{0}+\sum_{s}d_{s}q_{s}\right)+\theta_{r}\;v=-\Gamma_{r}\;{\ddot{w}}_{B},$$

(5) $$C_{p}\dot{v}+\frac{v}{R}-\sum_{r}\theta_{r}{\dot{q}}_{r}=0,$$

per electrical port, where *Γ_r_* is the base-excitation participation, *θ_r_* the electromechanical coupling of the port in question, and

(6) $$d_{r}\;=\;\varphi_{r}^{a}\left(x_{m}\right)-\varphi_{r}^{b}\left(x_{m}\right)$$

is the differential participation of mode *r* at the element. The vector d = (*d*_1_,*d*_2_,…) is the single object through which the slit element sees the structure: the nonlinearity enters mode *r* with weight *d_r_* and is driven by the *d*-weighted combination of all modal coordinates. Throughout, a tilde marks a quantity re-evaluated in the eigenbasis that diagonalizes the *k*_1_-shifted linear operator of Section 2.5 (the transformation itself is written out at the start of Section 2.7; e.g., ${\tilde{\omega }}_{r}$, ${\tilde{d}}_{r}$, ${\tilde{f}}_{r}={\tilde{\omega }}_{r}/\left(2\;\pi \right)$); the untitled symbols *ω_r_*, *d_r_*, *f_r_* used here and in Section 2.3 are the bare linear-branched-beam values with no element stiffness applied.

### 2.3. The Exact Mode-Selectivity Theorem

**Proposition** **1.** (exact selectivity at prong symmetry)**.** *If* M_1_ = M_2_*, the structure possesses a mirror symmetry about the slit centreline. Its modes separate into a symmetric family* 𝒮 *(*$\varphi_{r}^{a}=\varphi_{r}^{b}$*, root active) and an anti-symmetric family* 𝒜 *(*$\varphi_{r}^{a}=-\varphi_{r}^{b}$*,* $\varphi_{r}^{\left(1\right)}\equiv 0$*). Then* d_r_ = 0 *for every* r ∈ 𝒮*, and the slit element is absent from the symmetric-mode equations at every order in the response amplitude.*

The proof is immediate from Equation (6) and the junction conditions of the branched model: for *r* ∈ 𝒜, the moment and shear balances at the junction have identically zero right-hand sides, forcing the root field to vanish with the clamped conditions. Two corollaries carry the design weight. Blind mode*:* Anti-symmetric modes have *Γ_r_* = 0 and $\theta_{r}^{\Sigma }=0$ exactly: they are neither excited by uniform base acceleration nor read by the shared electrode, the exact-symmetry limit of the weak second peak reported in Ref. [31]. No self-pumping: At *μ* = 0, no amount of slit nonlinearity transfers energy from the symmetric to the anti-symmetric family. In-phase motion produces Δ≡0; thus, the element never deforms. Intermodal transfer requires prong mistuning, which is precisely what makes the coupling a design variable rather than an accident. 

### 2.4. Mistuning as the Coupling Control

Let $M_{1,2}=\bar{M}\left(1\pm \mu \right).$ First-order eigenvector perturbation about the symmetric configuration mixes a small amount of the anti-symmetric mode into the symmetric one with amplitude

(7) $$c=\chi \mu ,\;\;\chi =\frac{2\bar{M}\;\omega_{1}^{2}\;\varphi_{1}^{S}\left(L\right)\;\varphi_{2}^{A}\left(L\right)}{\omega_{2}^{2}-\omega_{1}^{2}},$$

where $\varphi_{1}^{S}\left(L\right)$ is the symmetric-mode tip deflection and $\varphi_{2}^{A}\left(L\right)$ is the anti-symmetric-mode prong-*a* tip deflection, so that the first mode acquires the differential participation

*d*_1_ = *χ* *μ* *d*_2_. (8)

Equation (8) is confirmed against the exact eigensolution with an error of 0.05% at *μ* = 0.05 (Section 3). The mistuning *μ* is therefore a linear dial on the strength with which the fundamental mode feels the slit element.

### 2.5. Rank-One Linear Lever and Differential Bistability

The linear part of the element enters the modal stiffness as the rank-one update $\mathrm{diag}\left(\omega_{r}^{2}\right)+k_{1}\;dd^{⊤}$. At *μ* = 0, the update lives entirely in the anti-symmetric block: *f*_1_ is exactly invariant, while

(9) $${\tilde{\omega }}_{2}^{2}=\omega_{2}^{2}+k_{1}d_{2}^{2}$$

to two-mode order. The anti-symmetric family softens to instability at

(10) $$k_{1}^{\mathrm{cr}}=-{\left[\sum_{r\in A}d_{r}^{2}/\omega_{r}^{2}\right]}^{-1},$$

whose two-mode leading term $-\omega_{2}^{2}/d_{2}^{2}$ is 0.2% away for the present design. Beyond $k_{1}^{\mathrm{cr}}$, the straight-slit equilibrium loses stability through a supercritical pitchfork in Δ alone: the harvester becomes bistable in its differential coordinate while remaining monostable in its common coordinate, a regime in which the in-phase mode continues to behave as the validated linear harvester, while the prong pair buckles into either of two states ±Δ_eq_.

### 2.6. Internal-Resonance Classification and the Coupling Constant

With mistuning on, the quadratic slit force generates the resonant pair of terms

(11) $${\ddot{q}}_{1}+\cdots +2\Lambda \;q_{1}q_{2}=\cdots ,\;\;{\ddot{q}}_{2}+\cdots +\Lambda \;q_{1}^{2}=\cdots ,\;\;\Lambda =k_{2}\;{\tilde{d}}_{1}^{2}\;{\tilde{d}}_{2},$$

where tildes denote evaluation in the basis that diagonalises the *k*_1_-shifted linear part (Section 2.7). By Equation (8), $\Lambda \propto k_{2}\;\chi^{2}\mu^{2}d_{2}^{3}$: the 2:1 channel is gated by two independent symmetry breakings: the mounting offset Δ_0_ (which creates *k*_2_) and the mass mistuning *μ* (which creates *d*_1_). Table 1 summarises the accessible regimes; the 1:3 interaction, driven by the cubic *k*_3_, requires only *μ* ≠ 0 and no offset, with the self- and cross-Duffing monomials retained in the amplitude equations.

### 2.7. Modulation Equations: The Two Excitation Cases

All quantities below carry tildes: they are evaluated after diagonalising the linear part, including the element stiffness, $\mathrm{diag}\left(\omega_{r}^{2}\right)+k_{1}dd^{⊤}=T\mathrm{diag}\left({\tilde{\omega }}_{r}^{2}\right)T^{⊤}$, with $\tilde{d}=T^{⊤}d$, $\tilde{\Gamma }=T^{⊤}\Gamma$, and the modal damping ratios *ζ_r_* defined on these shifted modes; since *k*_1_ moves ${\tilde{\omega }}_{2}$ by tens of percent, assigning damping to the unshifted modes mis-scales the response by the same fraction. Writing ${\tilde{\omega }}_{2}=2{\tilde{\omega }}_{1}+\varepsilon \sigma_{1}$ and applying the method of multiple scales [44] yields two qualitatively different responses.

#### 2.7.1. Case A: Drive near the Anti-Symmetric Mode, $\Omega ={\tilde{\omega }}_{2}+\varepsilon \sigma_{2}$

The slow flow

(12) $$a_{1}\prime =-\zeta_{1}{\tilde{\omega }}_{1}a_{1}-\frac{\Lambda }{2{\tilde{\omega }}_{1}}a_{1}a_{2}\sin\gamma_{1}$$

(13) $$a_{2}\prime =-\zeta_{2}{\tilde{\omega }}_{2}a_{2}+\frac{\Lambda }{4{\tilde{\omega }}_{2}}a_{1}^{2}\sin\gamma_{1}+\frac{f}{2{\tilde{\omega }}_{2}}\sin\gamma_{2},\;\;f=\left|{\tilde{\Gamma }}_{2}\right|A$$

admits a trivial (linear) branch and a coupled branch on which the driven mode saturates,

(14) $$a_{2}^{\mathrm{sat}}=\frac{{\tilde{\omega }}_{1}}{\Lambda }\sqrt{4\zeta_{1}^{2}{\tilde{\omega }}_{1}^{2}+{\left(\sigma_{1}+\sigma_{2}\right)}^{2}}\;\overset{\;\sigma_{1,2}=0\;}{\to }\;\frac{2\zeta_{1}{\tilde{\omega }}_{1}^{2}}{\Lambda },$$

independent of forcing, while the surplus spills into the subharmonic response *a*_1_ at *Ω*/2. The coupled branch activates at the base-acceleration threshold

(15) $$A_{\mathrm{thr}}=\frac{2{\tilde{\omega }}_{1}{\tilde{\omega }}_{2}}{\Lambda \;\left|{\tilde{\Gamma }}_{2}\right|}\sqrt{\zeta_{2}^{2}{\tilde{\omega }}_{2}^{2}+\sigma_{2}^{2}}\;\sqrt{4\zeta_{1}^{2}{\tilde{\omega }}_{1}^{2}+{\left(\sigma_{1}+\sigma_{2}\right)}^{2}}\;\overset{\;\sigma_{1,2}=0\;}{\to }\;\frac{4\zeta_{1}\zeta_{2}\;{\tilde{\omega }}_{1}^{2}{\tilde{\omega }}_{2}^{2}}{\Lambda \;\left|{\tilde{\Gamma }}_{2}\right|}.$$

Because *Λ* ∝ *μ*^2^ and $\left|{\tilde{\Gamma }}_{2}\right|\propto \mu$, the activation law is *A*_thr_ ∝ *μ*^−3^. At 2*A*_thr_ and perfect tuning, the subharmonic growth rate takes the universal value $\zeta_{1}{\tilde{\omega }}_{1}$.

#### 2.7.2. Case B: Drive near the Symmetric Mode, $\Omega ={\tilde{\omega }}_{1}+\varepsilon \sigma_{2}$

The anti-symmetric mode is driven only parametrically by $a_{1}^{2}$ and responds without threshold:

(16) $$a_{2}=\frac{\Lambda \;a_{1}^{2}}{4{\tilde{\omega }}_{2}\sqrt{\zeta_{2}^{2}{\tilde{\omega }}_{2}^{2}+{\left(2\sigma_{2}-\sigma_{1}\right)}^{2}}}.$$

The device then outputs at 2*Ω* on the differential port while driven by a single tone at $\Omega ≃{\tilde{\omega }}_{1}$: it manufactures its own second harmonic, a capability the linear device lacked (Appendix A), where the second peak required a second source line. The threshold and saturation phenomena of Equations (14) and (15) belong exclusively to Case A; Case B is a continuous quadratic transfer. This division mirrors the classical saturation-controller architecture of Oueini, Nayfeh and Pratt [43], here realised passively inside a monolithic harvester.

### 2.8. Validity of the Quadratic Closure

Two second-order effects bound the regime in which Equations (14) and (15) are quantitative, and both convert into design rules. (i) Parasitic self-quadratic. The monomial $\alpha_{22}q_{2}^{2}$ with $\alpha_{22}=k_{2}{\tilde{d}}_{2}^{3}$ softens the anti-symmetric mode at second order with dimensionless strength

(17) $$P=\frac{\alpha_{22}a_{2}^{\mathrm{sat}}}{{\tilde{\omega }}_{2}^{2}}=2\zeta_{1}{\left(\frac{{\tilde{\omega }}_{1}}{{\tilde{\omega }}_{2}}\right)}^{\;2}{\left(\frac{{\tilde{d}}_{2}}{{\tilde{d}}_{1}}\right)}^{\;2},$$

which is independent of the element strength: no magnet choice fixes it, because $a_{2}^{\mathrm{sat}}\propto 1/k_{2}$ cancels *α*_22_ ∝ *k*_2_. The only lever is the mode-shape ratio, i.e., the mistuning itself: clean autoparametric operation requires strong prong asymmetry (*P* = 0.28 at *μ* = 0.5, closure visibly broken; *P* = 0.057 at *μ* = 0.8, closure quantitative). (ii) Cubic detuning. The Duffing shift of ${\tilde{\omega }}_{2}$ is proportional to $k_{3}/k_{2}^{2}$ at fixed saturation amplitude; for the dipole element, this ratio is a pure shape function of the offset and possesses an exact zero at

(18) $$\Delta_{0}^{⋆}≃0.246\;g_{0},$$

the root of $U_{s}^{\left(4\right)}$. Mounting at $\Delta_{0}^{⋆}$ annihilates the cubic coefficient identically and removes the detuning by construction.

## 3. Computational Methods and Verification

### 3.1. Exact Branched Eigen Solver and Two-Port Electromechanics

The linear branched modes are computed exactly: cosine/sine/hyperbolic shape functions on the root and each prong, assembled into a 12 × 12 characteristic system enforcing the clamp, continuity of displacement and slope at the junction, junction moment and shear balance with the width weights *b*, *b_p_*, and tip conditions carrying *M*_1_, *M*_2_. Explicitly, the twelve conditions are as follows: (1–2) clamp at the root, *w*_1_(0) = 0, *w*_1_′(0) = 0; (3–6) continuity of displacement and slope at the junction *x* = *L*_1_, *w*_1_ = *w_a_*, *w*_1_ = *w_b_*, *w*_1_′ = *w_a_*′, *w*_1_′ = *w_b_*′; (7) junction moment balance (the common per-width stiffness $\hat{\mathrm{EI}}$ cancels), *b w*_1_″ = *b_p_*(*w_a_*″ + *w_b_*″); (8) junction shear balance, *b w*_1_‴ = *b_p_*(*w_a_*‴ + *w_b_*‴); (9–10) free tip of prong *a* (mass *M*_1_, at *x* = *L*), moment-free *w_a_*″(*L*) = 0 and shear balancing the tip-mass inertia, $\hat{\mathrm{EI}}\;b_{p}\;w_{a}\prime \prime \prime \left(L\right)=-\omega^{2}M_{1}\;w_{a}\left(L\right)$; (11–12) free tip of prong *b* (mass *M*_2_), *w_b_*″(*L*) = 0 and $\hat{\mathrm{EI}}\;b_{p}\;w_{b}\prime \prime \prime \left(L\right)=-\omega^{2}M_{2}\;w_{b}\left(L\right)$. Roots of the row-normalised determinant give the frequencies; the SVD null vector gives the fields, mass-normalised, including the tip masses. From the fields, the solver evaluates *Γ_r_*, the port couplings $\theta_{r}^{\Sigma },\theta_{r}^{\Delta }$, the differential participations *d_r_*, and the mean-tip participations. Two-port frequency response functions follow from the modal system Equations (4) and (5) with matched resistive loads on each port.

### 3.2. Geometry Calibration and Its Declared Limits

**Figure A1:**
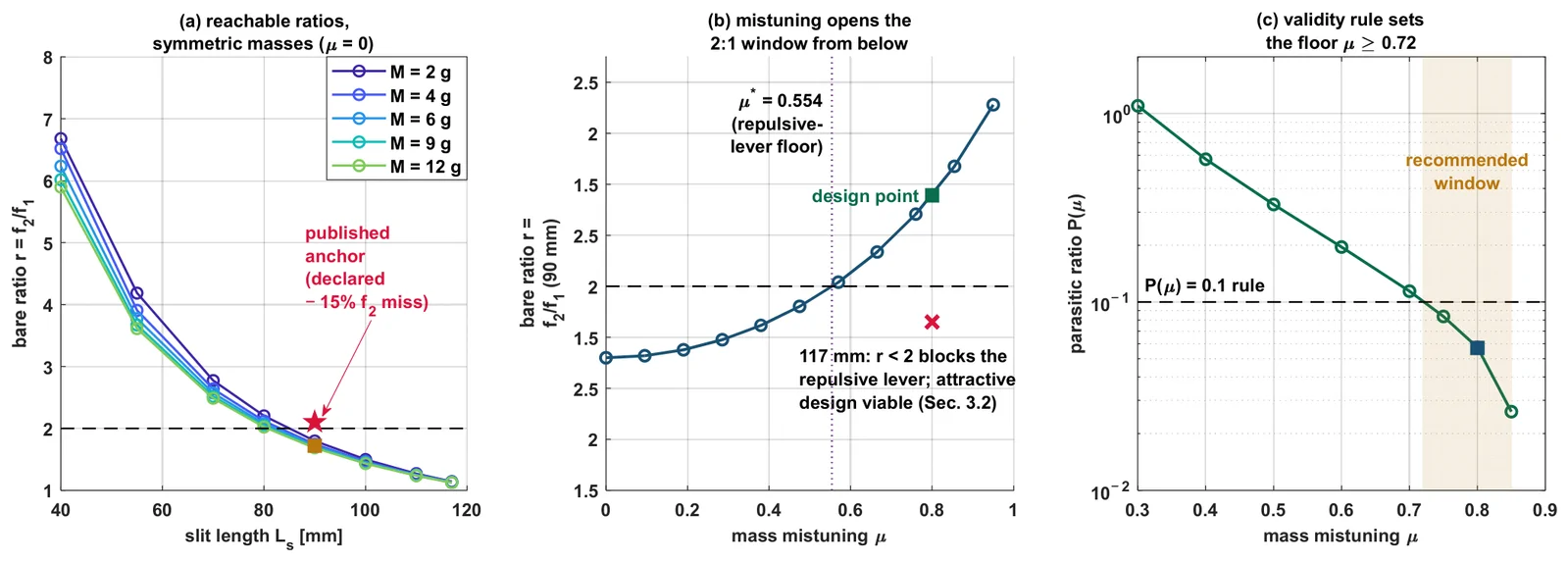
Reachability and the mistuning window (calibrated model). (**a**) Bare ratio *r* = *f*_2_/*f*_1_ over slit length for symmetric tip masses; the dashed line is r = 2, the 2:1 condition; the model point at the 90 mm, 6 g case (square) against the published anchor (star) displays the declared −15% anti-symmetric calibration miss. (**b**) *r*(*μ*) at 90 mm: the repulsive-lever floor *μ*^⋆^ = 0.554; (dotted vertical line); the design point, and the 117 mm geometry’s *r*(0.8) = 1.86 < 2 (cross) that excludes it from 2:1 operation; the dashed line is again r = 2. (**c**) Parasitic ratio 𝒫(*μ*) with the 𝒫 = 0.1 rule (dashed line) and the recommended window (shaded).

**Figure A2:**
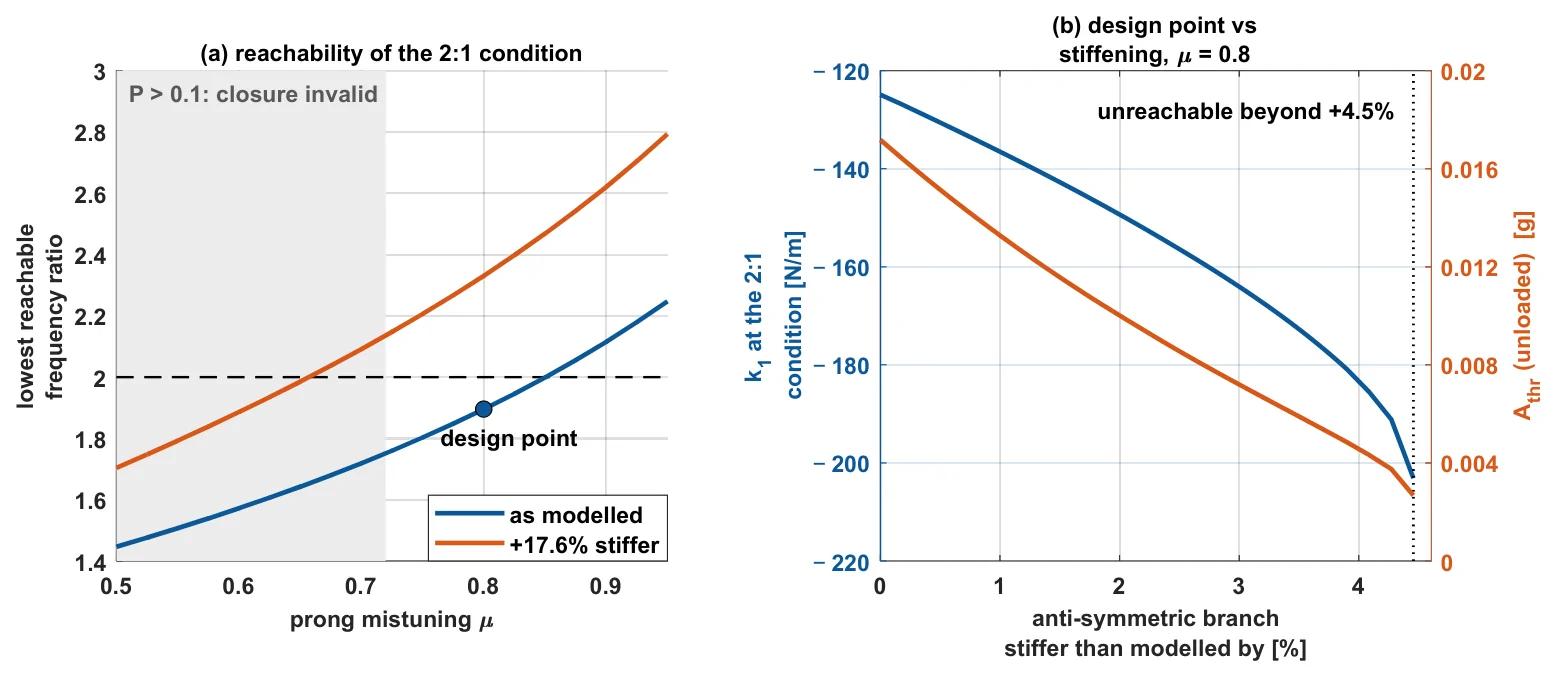
Sensitivity of the 2:1 design point to the declared anti-symmetric calibration miss (Section 3.2). (**a**) Lowest ratio ${\tilde{f}}_{2}/{\tilde{f}}_{1}$ the repulsive lever can reach at each mistuning *μ* (minimum over $k_{1}\in (k_{1}^{\mathrm{cr}},0]$), for the branch as modelled and for a branch 17.6% stiffer (the full declared miss); the 2:1 condition is reachable only where a curve lies below 2—up to *μ* = 0.85 as modelled, but only below *μ* = 0.66 at the full miss, inside the region excluded by the 𝒫 ≤ 0.1 rule (shaded). (**b**) Re-tuned *k*_1_ and unloaded threshold *A*_thr_ at *μ* = 0.8 against the stiffening of the branch; beyond +4.5% the condition cannot be met.

The published anchors of the linear device [31] are used to reconstruct a working geometry: the uniform-beam limit pins $\sqrt{\hat{\mathrm{EI}}/\hat{m}}/L^{2}$ (41.8 Hz), and a two-parameter solve (laminate thickness scale and width *b*) fits the 117 mm-slit, 5 g-tip-mass pair *f*_1,2_ = (27.15,45.66) Hz exactly, giving *h_s_* = 0.715 mm, *h_p_* = 0.357 mm, *b* = 43.3 mm, *L* = 126.3 mm. The reconstruction then predicts the 10 g case within −4.4%/+1.9% and the symmetric-mass 90 mm-slit case within +3.5% on *f*_1_ but −15% on *f*_2_: the four published anchors overdetermine the branched Euler–Bernoulli model, and we state this openly. All structural theorems of Section 2 are geometry-independent identities verified to machine precision; the quantitative design-point numbers (*A*_thr_, magnet sizes) are illustrative pending recalibration to the fabricated geometry. “Illustrative” licences order-of-magnitude and scaling statements and the complete branch topology; it does not licence transferring the quoted decimals to hardware. Propagating the declared anti-symmetric-branch uncertainty as a ±15% frequency-scale factor *λ* (to which *A*_thr_ ∝ *λ*^4^ under Equation (15)) brackets the headline threshold as *A*_thr_ ∈ [0.009, 0.030] g, with quoted powers scaling as *λ*^2^ at fixed drive. The bracket convention is explicit: the measured anchor is taken as truth and the beam model’s −15% as the model error; the plate cross-check demonstrates the branch’s model-form sensitivity rather than supplying a second truth. (Using instead the full beam↔plate↔anchor spread, *λ* ∈ [0.84,1.18], gives [0.009, 0.033] g essentially the same interval, which is why the ±15% convention is retained). That bracket assumes the 2:1 tuning survives, as it does under a uniform frequency scaling; the declared miss is not uniform (*f*_1_ within +3.5%, *f*_2_ −15%), and its non-uniform part is the more consequential one. Re-tuning the model with the anti-symmetric branch alone stiffened shows by how much: at +2%, the 2:1 point moves from *k*_1_ = −125 to −149 N/m and *A*_thr_ falls from 0.017 to 0.010 g; beyond +4.5%, the repulsive lever can no longer bring ${\tilde{f}}_{2}/{\tilde{f}}_{1}$ down to 2 at *μ* = 0.8, because the veering that the mistuning creates floors the achievable ratio (1.90 at the design point, 2.33 at the full +17.6% miss; Figure A2). At the full miss, the ratio is reachable only for *μ* ≲ 0.66, below the validity floor *μ* ≥ 0.72 (Figure A1c). Should the fabricated branch prove that stiff, the 90 mm repulsive design point is not shifted but replaced by a re-tuned slit length (Appendix A) or by the attractive 117 mm design below, while the theorems, scaling laws, and closed forms are untouched. The 90 mm slit is retained as the design point on merit, not necessity. At the exactly anchored 117 mm geometry, the bare ratio at *μ* = 0.8 is *r* = 1.86 < 2 (Appendix A), so the repulsive lever cannot reach 2:1 there, but the attractive polarity can. That design was computed rather than excluded: *k*_1_ = +62.5 N/m (a magnet pair of ≈0.7× the reference moment, cube side ≈ 0.9×), ${\tilde{f}}_{1,2}=\left(21.9,\;43.9\right)$ Hz, parasitic ratio 𝒫 = 0.073 (within the validity rule), and a gap-axis attraction of 0.16 N whose static closure is 3.3 μm of the 5 mm gap with a pull-in margin above 500×. This is mechanically unobjectionable. Its activation threshold, however, is *A*_thr_ = 0.058 g unloaded, 3.4× the 90 mm design’s 0.017 g, because |*Λ*| is 2.9× smaller; at the 0.05 g reference ambient, it would not activate even unloaded. The 90 mm point is therefore kept for its threshold performance, and the attractive 117 mm design stands as the calibration-robust alternative for stronger ambient.

### 3.3. Verification Battery

Table 2 condenses the seven-stage verification. The multiple-scales results were additionally re-derived symbolically from the secular conditions, and the slow-flow fixed points, stability, and closed-form Equations (14) and (15) were confirmed against direct integration of the full cubic reduced model with consistently transformed damping.

### 3.4. Independent Finite-Element Cross-Check

Every entry of Table 2 verifies the model against itself; to bound model error rather than coding error, the reconstructed geometry was additionally analysed with an independent numerical family: a two-dimensional Kirchhoff plate model (*ν* = 0.33, full twist energy, consistent mass) discretised with Morley finite elements on a mesh containing the physical slit, so the prongs are kinematically independent and the tip masses load the prong tip edges. This model shares neither the kinematic ansatz nor the discretisation of the branched Euler–Bernoulli eigensolver. Table 3 reports the comparison. The structural content of Proposition 1 is reproduced independently: at *μ* = 0, the symmetric family’s differential participations are zero to 8 × 10^−5^ relative and the anti-symmetric family’s base participations to 9 × 10^−5^discretization-asymmetry level and the participation magnitudes *d*_2_ agree between families to 2 − 4%, with *f*_1_ within 2% and the *μ*-gating of *d*_1_ reproduced (*d*_1_(0.4)/*d*_1_(0.8) of 0.63 beam vs. 0.72 plate, both showing the same finite-*μ* softening of the linear law). The anti-symmetric frequencies, by contrast, differ by −15.6% between the families (the plate prongs are chordwise compliant, where the beam model is rigid). The direction matters and is stated plainly: the measured 90 mm anchor sits ∼15% above the beam model, while the plate sits ∼15% below it, so the plate result does not explain the anchor miss causally the fabricated device is stiffer on this branch than either idealisation, for reasons neither model contains. Of the three candidates, one is already quantitatively bounded and a second admits an order-of-magnitude check. Chordwise compliance of the tip-mass blocks is precisely what the plate model relaxes, and its effect is exactly the measured −15.6% of Table 3, but of the wrong sign to explain the miss, since chordwise compliance can only soften the branch further, not stiffen it. Clamp and adhesive stiffening acts, to leading order, as an outward shift of the effective clamp point that shortens the free prong length; at this symmetric-mass point, the 6 g tip mass carries about three-quarters of the prong’s effective inertia (the laminate prong itself is ≈8.7 g distributed, ≈2 g referred to the tip), so *f*_2_ scales close to the tip-mass limit *f*_2_ ∝ *L*^−3/2^, and the branched-beam model itself gives the figure directly: closing the full +17.6% gap ($f_{2}^{\mathrm{meas}}/f_{2}^{\mathrm{model}}$ = 1/0.85) by this mechanism alone would require moving the effective clamp outward by ≈8.6 mm, i.e., shortening the 90 mm prong by ≈10% at the slit root, an order of magnitude larger than a realistic fillet or adhesive bond-line (sub-millimetre to ~1–2 mm) can supply, which bounds this candidate to a minor contributor. Kirchhoff underestimation of the laminate is not independently testable without a higher-order (e.g., Mindlin or layer-wise) laminate model, beyond the present scope. None of the three, at a physically realistic magnitude, closes the gap on its own, so the discrepancy is left declared rather than adjusted away. What the cross-check legitimately establishes is therefore twofold: (i) the selection rules and participations are model-form robust—the theorem’s content survives the change of family untouched; and (ii) the anti-symmetric branch’s frequency is strongly model-form sensitive (a 15.6% beam ↔ plate spread), consistent with that branch carrying the calibration miss while leaving its physical origin an open question for the experimental programme of Section 6.

## 4. Results and Discussion

### 4.1. The Linear Regime: An f_*2*_ Lever with f_*1*_ Pinned at μ = 0

**Figure 2:**
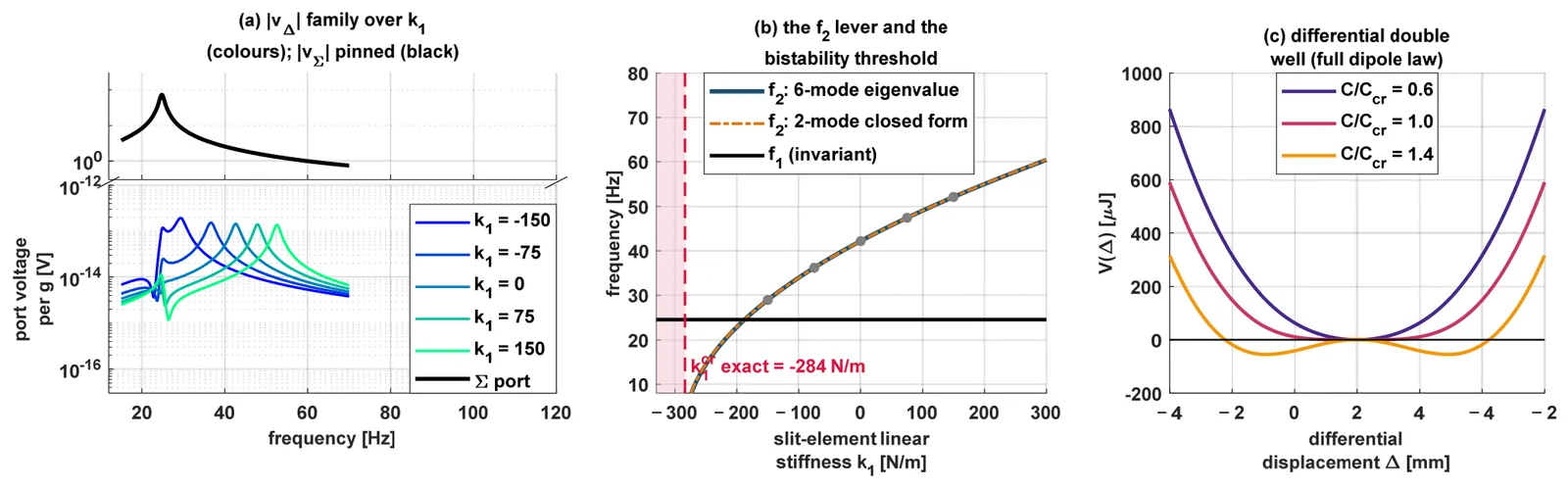
Linear regime of the slit element (symmetric masses). (**a**) Two-port FRF family: |*v*_Δ_| Sweeps over tens of hertz with *k*_1,_ while |*v*_Σ_| is exactly pinned at *f*_1_. (**b**) The *f*_2_ lever: six-mode eigenvalue vs. the two-mode closed form, with the exact rank-one bistability threshold. (**c**) Differential double-well potential from the full dipole law; he black horizontal line marks V = 0, the potential at Δ = 0, from which the well depths are read; |*C_cr_*| corresponds to a ∼3 mm N42 cube pair at a 5 mm gap. (**a**)’s *y*-axis is broken to separate the widely different amplitude ranges of the Σ and Δ ports. At μ = 0, the Δ port is reached only through participations that vanish by mirror symmetry (the anti-symmetric family has zero base participation and the symmetric family zero Δ-port coupling; both are zero to 10^−16^ here), so the |vΔ| levels of 10^−14^–10^−13^ V/g are numerical residue: only the resonance locations, which sweep with k_1_, are physical.

Figure 2a shows the two-port FRF family at *μ* = 0 as the element stiffness sweeps *k*_1_ ∈ [−150,+150] N/m. The differential-port resonance slides from 33 to 60 Hz a full octave of authority from a millimetre-scale magnet pair, while the sum-port resonance does not move by so much as 10^−10^ Hz. The physics is the selectivity theorem made visible: the element acts on Δ, every symmetric mode has Δ-participation exactly zero, and so the power-carrying resonance is not merely weakly perturbed but algebraically absent from the element’s reach. Panel (b) quantifies the lever: the six-mode eigenvalue and the two-mode closed form Equation (9) are indistinguishable to 22 mHz, and the exact rank-one threshold Equation (10) ($k_{1}^{\mathrm{cr}}=-284$ N/m for the symmetric-mass configuration of this subsection; the *μ* = 0.8 design point has −286.8 N/m, Table 4) bounds the softening branch. For a practitioner, this completes the inverse design the linear device could not close: choose masses and slit for *f*_1_, then place *f*_2_ afterwards and independently with the element—attractive polarity to raise it, repulsive to lower it.

### 4.2. Static Bifurcation: Buckling of the Prong Pair, Not the Beam

**Figure 3:**
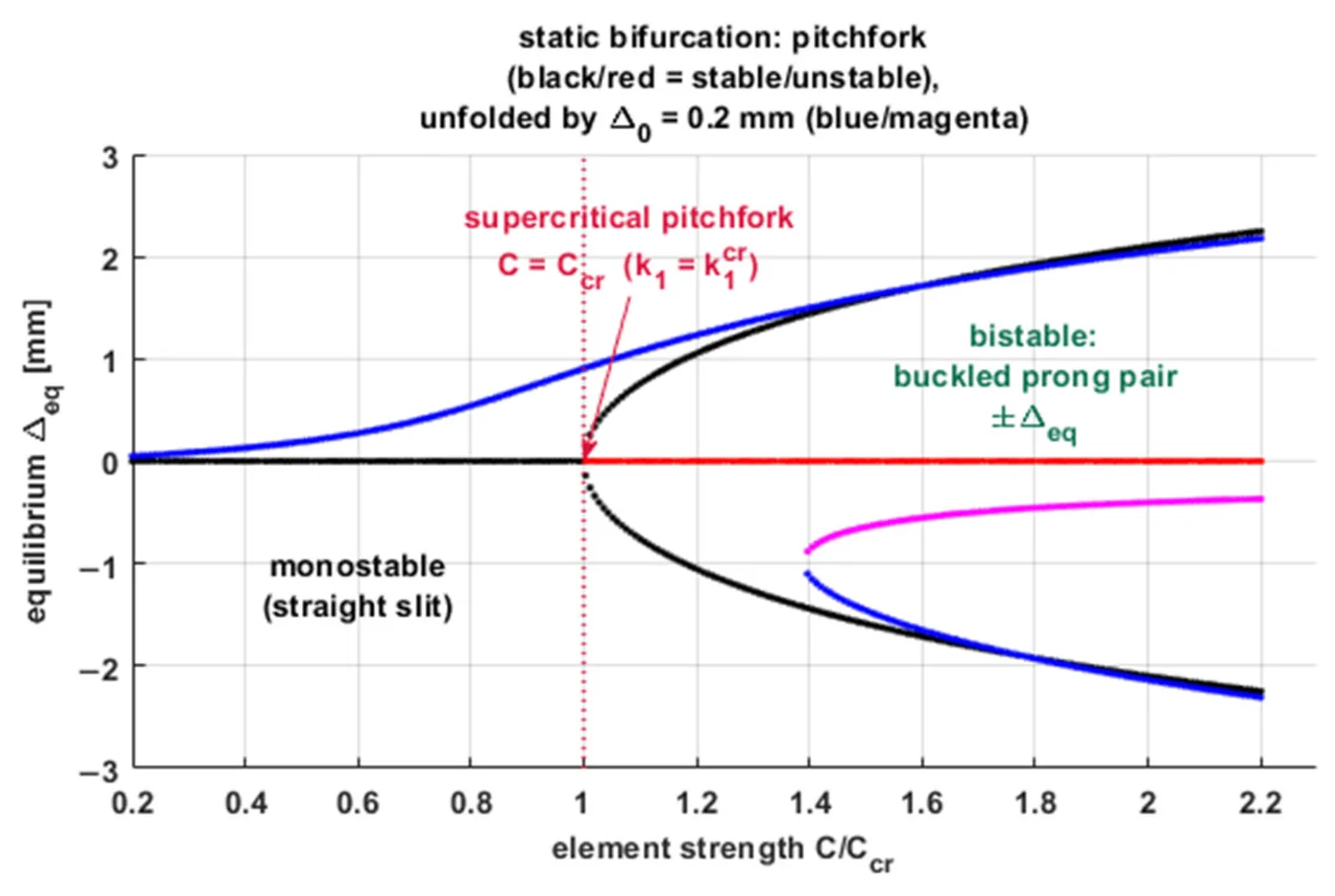
Static bifurcation of the differential coordinate. Supercritical pitchfork at *C* = *C_cr_* ($k_{1}=k_{1}^{\mathrm{cr}}$); a 0.2 mm, mounting imperfection unfolds it into a fold with hysteresis.

Beyond $k_{1}^{\mathrm{cr}}$ (repulsive element strengthened past *C_cr_*), the straight-slit state destabilises through the supercritical pitchfork of Figure 3, computed with the exact dipole law rather than its truncation. The equilibria ±Δ_eq_ are differential buckled states: the two prongs bow apart (or together), while the beam remains straight and its in-phase dynamics remain those of the validated linear harvester. This is the essential contrast with piezomagnetoelastic bistability [9,11], where the double well lives on the same coordinate that carries the primary response and therefore reshapes it. Here, bistability and linear operation coexist on orthogonal coordinates. A mounting imperfection Δ_0_ = 0.2 mm unfolds the pitchfork into a fold with hysteresis (dotted branches), quantifying the assembly tolerance within which the symmetric picture survives.

### 4.3. Symmetry-Breaking Laws: How the Coupling Channel Opens

**Figure 4:**
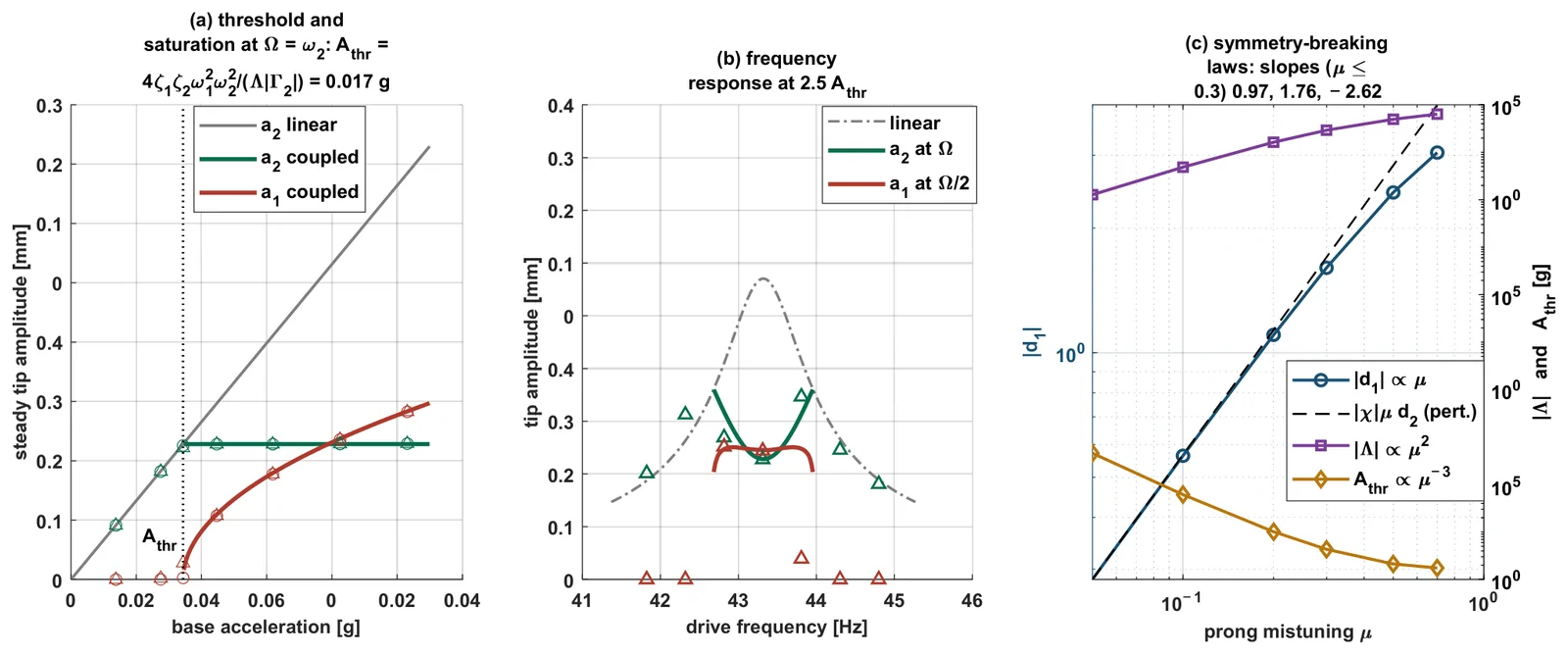
Two-to-one internal resonance at the design point (slit 90 mm, *M*_1_/*M*_2_ = 10.8/1.2 g, element tuned to ${\tilde{f}}_{2}=2{\tilde{f}}_{1}$; *ζ*_1,2_ = 0.012). (**a**) Force response at $\Omega ={\tilde{\omega }}_{2}$: linear branch, activation at *A*_thr_ = 0.017 g, saturation of *a*_2_ at the forcing-independent plateau in Equation (14), and spillover into the subharmonic *a*_1_; open markers are direct integration of the full cubic model at every amplitude from two initial conditions: circles from rest (cold start), triangles seeded on the coupled branch at 1.2 a_2_ˢᵃᵗ, marker colors follow the curves, green for a_2_ (response at *Ω*) and red for a_1_ (response at *Ω*/2): below threshold, both settle onto the linear branch, and clear of it both converge to the same saturated state, which is the numerical test of the stability exchange. (**b**) Frequency response at 2.5*A*_thr_ (triangles: seeded direct integration). (**c**) Symmetry-breaking scaling laws with fitted slopes over *μ* ≤ 0.3; the departure of the fitted exponents from the asymptotic values 1,2,−3 is finite-*μ* curvature (mode localisation), quantified in the text.

Figure 4c tests the scaling architecture of Section 2.6. The exact differential participation of the first mode tracks the perturbation law *d*_1_ = *χμd*_2_ to 0.05% at small *μ* and departs only through mode localisation [49] beyond *μ*∼0.3; the coupling constant and activation threshold follow the predicted powers with fitted slopes 0.97 (*d*_1_, asymptotic exponent 1), 1.76 (*Λ*, exponent 2) and −2.62 (*A*_thr_, exponent −3) over *μ* ≤ 0.3; over the full range, the fits soften to 0.91, 1.50 and −2.15 as mode localisation saturates the laws, while the exact small-*μ* verification (0.05% at *μ* = 0.05) confirms the asymptotic exponents themselves. Physics deserves emphasis because it is the paper’s organising idea: the nonlinear coupling is not a material constant but a symmetry order parameter. At *μ* = 0, the channel is closed by an exact selection rule; the designer opens it continuously, and the cubic sensitivity of *A*_thr_ to *μ* means that a deliberate, strong mass asymmetry (the design point uses *μ* = 0.8, a 9:1 tip-mass ratio) converts an inaccessible threshold into a sub-0.1 g one. This contrasts with IR harvesters built on auxiliary oscillators [36,37] or global geometry [35], where the coupling strength is fixed by the architecture and cannot be dialled without retuning the commensurability itself; here, commensurability (element *k*_1_) and coupling strength (*μ*, Δ_0_ through *k*_2_) are independent knobs.

### 4.4. The 2:1 Internal Resonance: Threshold, Saturation, Spillover

Figure 4a is the central nonlinear result. Driving at $\Omega ={\tilde{\omega }}_{2}$, the response follows the linear branch until *A*_thr_ = 0.169 m/s ^2^ (0.017 g for this design point), where the trivial branch loses stability, and the coupled branch takes over: the driven anti-symmetric amplitude locks to the saturation plateau in Equation (14) flat to within 0.3% across a factor of three in drive, while every increment of input power is diverted into the subharmonic symmetric response at *Ω*/2. Direct integration of the full cubic model (markers) reproduces the linear branch, the threshold location, the plateau, and the spillover amplitudes to 0.1–2%; cold-start simulations below threshold decay to the linear branch and above threshold grow into the coupled one at the predicted universal rate $\zeta_{1}{\tilde{\omega }}_{1}$, confirming the stability exchange and not merely the fixed points.

The physics is an energy pump running in the protective direction. The quadratic element converts two quanta of the subharmonic mode into one of the driven modes and vice versa; on the coupled branch the phase locks so that the anti-symmetric mode, once it reaches the plateau, hands all surplus power to the symmetric mode, the mode wired to the robust, base-coupled, high-*θ* sum port. Saturation of this kind is classical in autoparametric absorbers [42,43] and has been exploited for bandwidth in IR harvesters [34]; what is new here is where it lives: the pump is monolithic, sits in a coordinate invisible to the primary response, and its threshold obeys an explicit symmetry-breaking law rather than an architectural accident. Two practical readings follow. First, the low measured threshold (0.017 g) puts activation well inside ambient-vibration territory, comparing favourably with the initiation thresholds that limit parametrically excited harvesters [18,19] and with the same root cause, since Equation (15) is the autoparametric analogue of the parametric activation condition. Second, the saturation plateau caps the differential tip excursion (0.11 mm here), i.e., the element self-limits: the magnet pair is protected from over-closure by the very dynamics it creates.

Figure 4b shows the frequency response at 2.5*A*_thr_: a coupled band of nearly constant *a*_2_ bounded by the two branch points, with the subharmonic forming the characteristic dome. The band, not the peak, is the harvesting resource within it that the device delivers simultaneously at *Ω* (differential port) and *Ω*/2 (sum port).

### 4.5. The Mounting Offset as a Lean Dial, and the Validity Map

**Figure 5:**
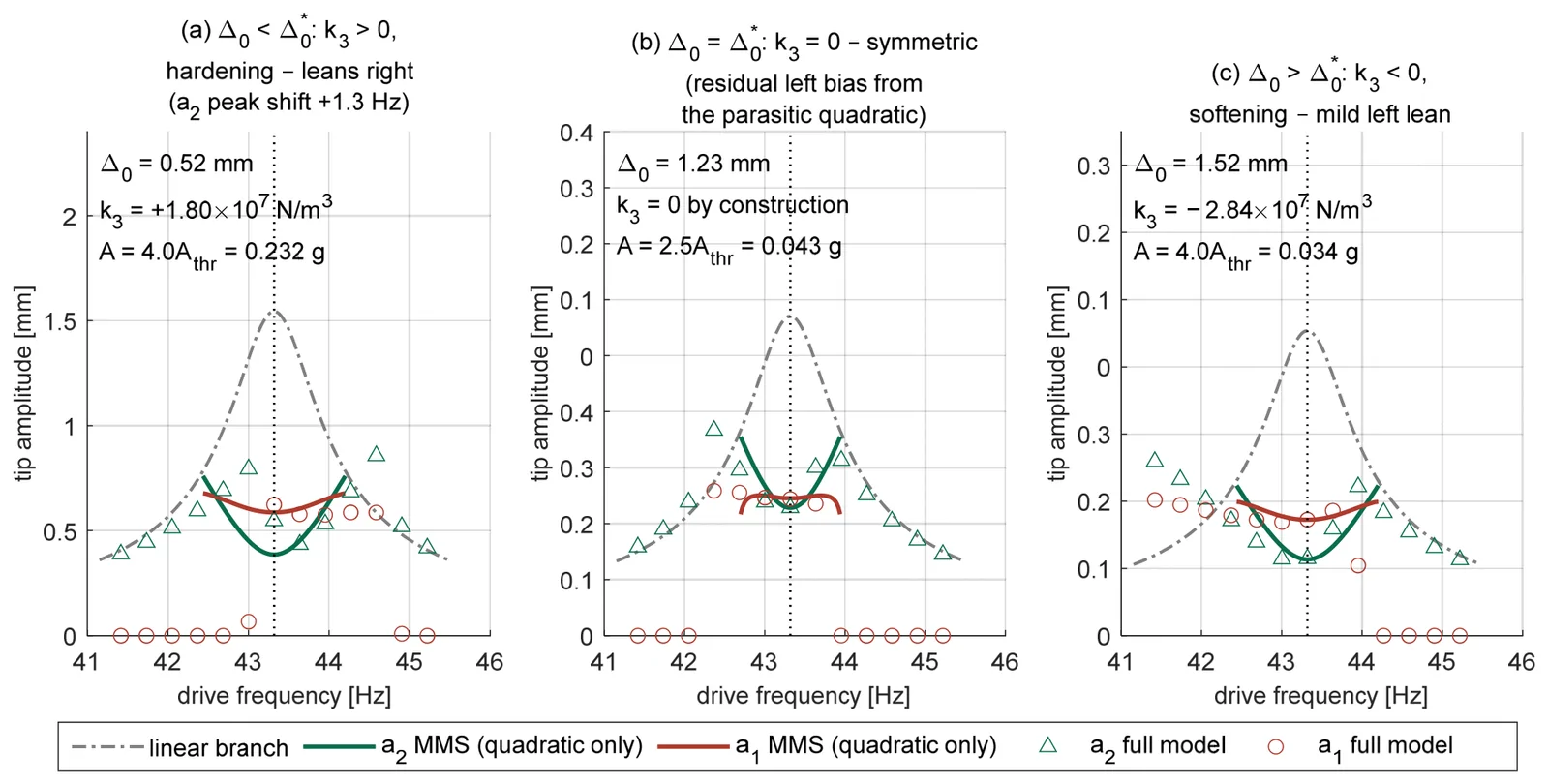
The mounting offset sets the lean of the coupled 2:1 response (drive near ${\tilde{f}}_{2}$; quadratic-only slow flow vs. direct integration of the full cubic model). (**a**) $\Delta_{0}<\Delta_{0}^{⋆}$: hardening, right lean. (**b**) $\Delta_{0}=\Delta_{0}^{⋆}$: symmetric, small residual left bias from the parasitic quadratic. (**c**) $\Delta_{0}>\Delta_{0}^{⋆}$: mild softening lean; the element is stronger, amplitudes smaller.

Figure 5 sweeps the coupled response for three mounting offsets. Below $\Delta_{0}^{⋆}$, the retained cubic is hardening, and the band leans right (measured *a*_2_ peak shift +1.3 Hz at Δ_0_ = 0.52 mm); at $\Delta_{0}^{⋆}=1.23$ mm, the response is symmetric to within a small left bias, the visible fingerprint of the parasitic quadratic Equation (17), resolved even by the direct simulations; above $\Delta_{0,}^{⋆}$ a mild softening lean appears, attenuated because the element is stronger there and the operating amplitudes correspondingly smaller. The asymmetry of the map itself is the design message: mis-mounting below $\Delta_{0}^{⋆}$ is harmful (lean plus threshold rise), mis-mounting above is nearly benign. Together with Equation (17), which forced the design point to strong mistuning (*μ* = 0.8, *P* = 0.057), these results bound the quadratic closure honestly: the elegant formulas Equations (14) and (15) are quantitatively precise inside the window that the two rules delimit and demonstrably fail outside it (*μ* = 0.5 trials showed the closure breaking at *P* = 0.28).

### 4.6. Bifurcation Structure: Activation Tongue, Branch Points, Hysteresis

**Figure 6:**
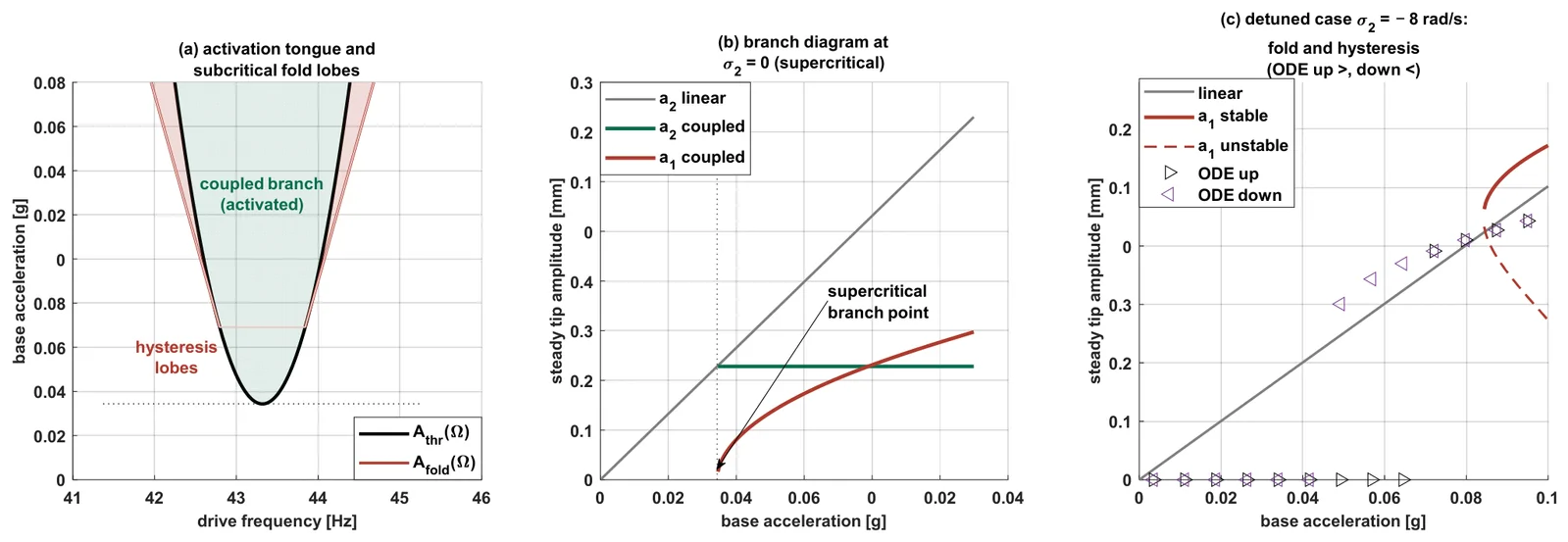
Bifurcation structure of the 2:1 internal resonance (*μ* = 0.8 design point). (**a**) Activation tongue *A*_thr_(*Ω*) with the subcritical fold lobes (shaded), where linear and coupled responses coexist. (**b**) Branch diagram at perfect tuning: supercritical branch point, no hysteresis. (**c**) Detuned case *σ*_2_ = −8 rad/s: fold and hysteresis; open markers are up (⊳) and down (⊲) sweeps of the full cubic model, confirming the predicted jumps.

Figure 6a assembles the dynamic bifurcation set of the 2:1 regime in the plane of drive frequency and base acceleration. The activation boundary *A*_thr_(*Ω*) of Equation (15) forms a V-shaped tongue touching its minimum (0.017 g) at $\Omega ={\tilde{\omega }}_{2}-$ the exact auto parametric analogue of the parametric instability tongues that govern parametrically excited harvesters [18]. Inside the tongue, the linear branch is unstable and the coupled branch is the unique attractor. Beyond the detuning $\left|\sigma_{2}\right|>\sqrt{2\zeta_{1}\zeta_{2}{\tilde{\omega }}_{1}{\tilde{\omega }}_{2}}$ (≈0.5 Hz here), the branch point turns subcritical: a fold curve *A*_fold_(*Ω*) peels away below the threshold curve, and in the lobes between the two, the linear and coupled responses *coexist*. Panels (b) and (c) show the corresponding branch diagrams. At perfect tuning, the transition is a clean supercritical branch point; amplitudes grow continuously from zero, with no jumps. At σ_2_ = −8 rad/s, the fold produces hysteresis, confirmed by up/down sweeps of the full model: the upward sweep jumps onto the coupled branch at A_thr_ (≈0.07 g), the downward sweep clings to it until the fold (≈0.045 g), and the enclosed loop is the usable memory of the device. The first-order slow-flow branch, drawn for reference, places the fold about 40% higher: at 2.4 linewidths of detuning, the quadratic-only closure is quantitative at the tongue’s centre and only qualitative on its flanks. The engineering readings are direct: sweep-down operation retains the coupled (power-favourable) state over a wider window than sweep-up; and the measured tongue minimum is the single most sensitive commissioning test of the entire model, since every constitutive parameter enters Equation (15).

### 4.7. Harvested Power Under Load: The Pump–Load Trade-Off

**Figure 7:**
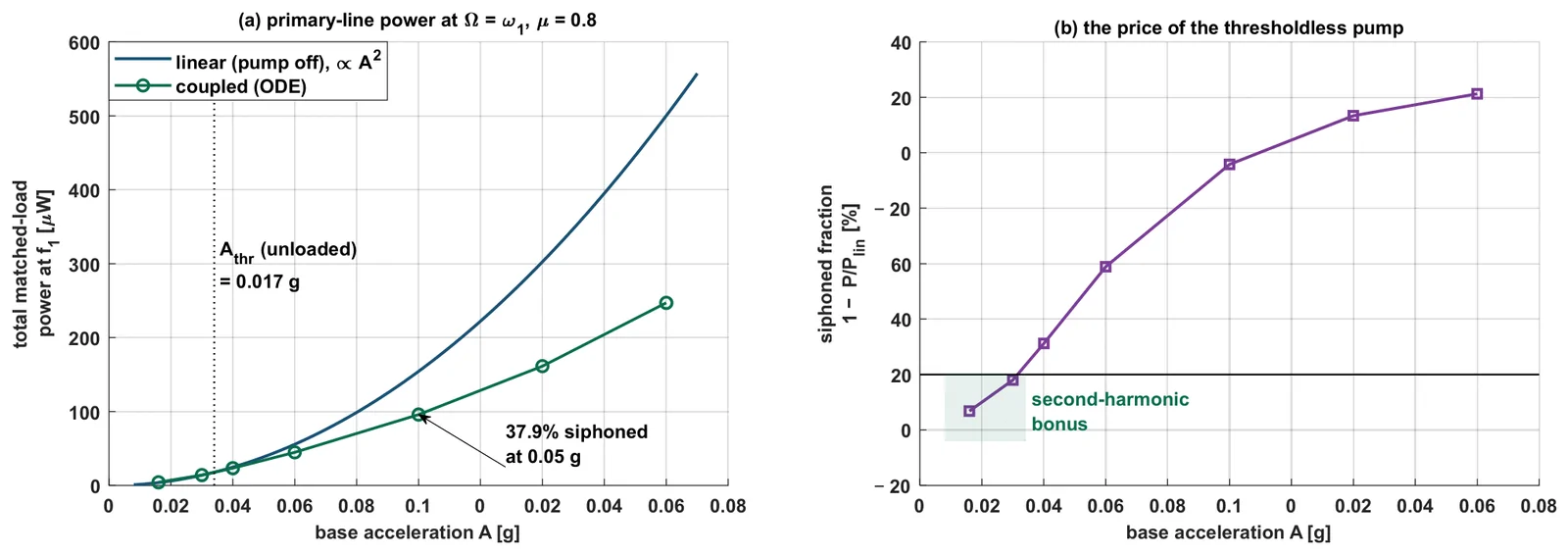
The price of the thresholdless pump at the *μ* = 0.8 design point, $\Omega ={\tilde{\omega }}_{1}$, matched load. (**a**) Total power against the linear branch (quadratic transfer off): below *A* ≃ 0.015 g, the second harmonic is a small net bonus; above it, back-action syphons the primary line (37.9% at 0.05 g). Circles: Direct integration of the full electromechanical model. (**b**) Syphoned fraction versus drive; the crossover sits near the unloaded *A*_thr_. Together with the 5.9% downshift of ${\tilde{f}}_{1}$ produced by the element at this *μ*, this panel is the quantitative price of pump capability, to be weighed against Figure 8.

**Figure 8:**
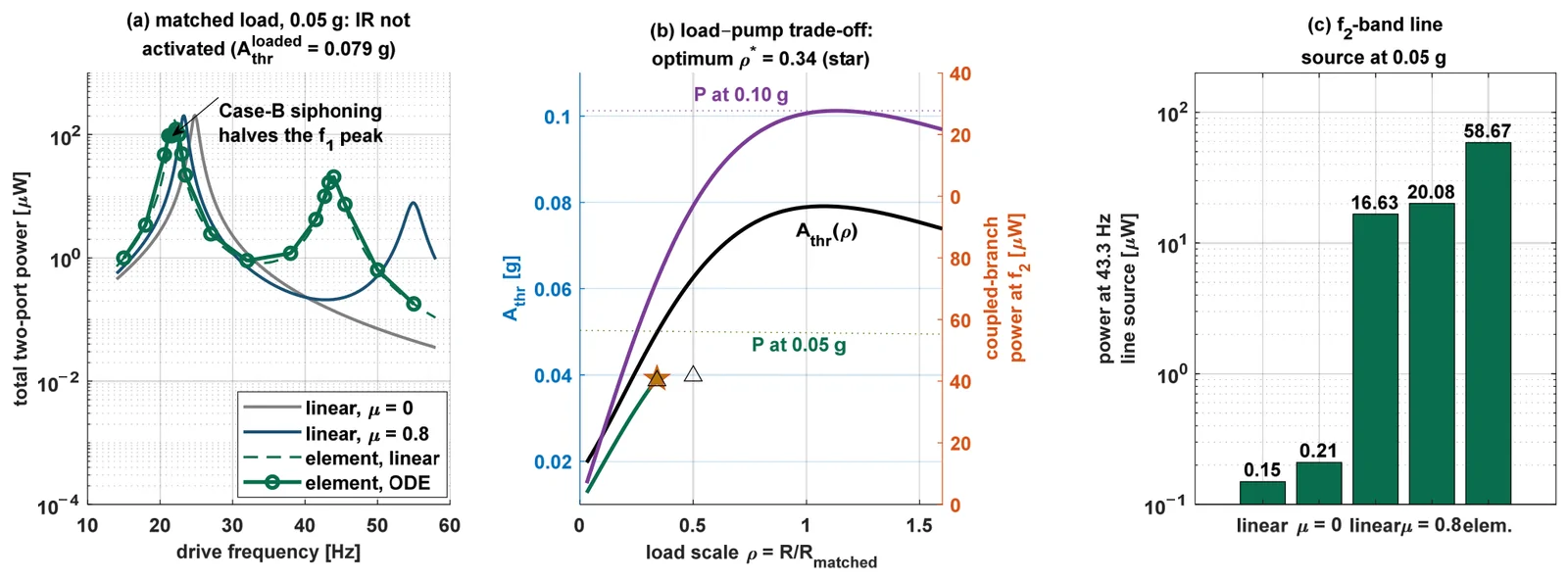
Power under load at 0.05 g. (**a**) Matched load: Linear baselines (exact two-port solutions) vs. the element device (full electromechanical simulations, circles); the internal resonance is not activated at this load ($A_{\mathrm{thr}}^{\mathrm{loaded}}=0.079$ g); swept integrals 354/352/448 μW⋅Hz. (**b**) The load–pump trade-off: threshold *A*_thr_(*ρ*) and coupled-branch power vs. load scale *ρ*; interior optimum *ρ*^⋆^ = 0.34; triangles are full-model validations (20.1 vs. 20.4 μW at *ρ*^⋆^). (**c**) Line source at 43.3 Hz, within-platform comparison plus the dedicated resonant benchmark (a plain cantilever length-tuned to the line, 58.7 μW): the lever converts a dead line into a served one; the pump adds 21% at this level.

Converting the dynamics into power exposes a coupling that the mechanical analysis alone conceals: the harvesting load damps the pump. Under matched two-port loads, the electrical contribution to the modal damping of this device is *ζ_e_*_,1_ = 0.0125 and *ζ_e_*_,2_ = 0.0150 comparable to the mechanical *ζ_m_* = 0.012 so the loaded activation threshold rises from 0.017 g to $A_{\mathrm{thr}}^{\mathrm{loaded}}=0.079$ g, and at a 0.05 g ambient the internal resonance does not activate at matched load (full simulations confirm: the subharmonic content at the band centre is ∼10^−11^, i.e., the loaded linear branch). Figure 7 and Figure 8a shows the resulting matched-load reality, with the nonlinear device represented entirely by full electromechanical simulations. Two honest effects compose the swept-band picture, and Figure 7 quantifies the first as a function of drive: the thresholdless Case-B transfer syphons the ${\tilde{f}}_{1}$ peak of the design point 95.9 against 154.4 μW for the same device with the quadratic transfer switched off, a 37.9% syphon at 0.05 g that grows to 51% by 0.09 g, while below *A* ≃ 0.015 g, the second harmonic is a small net bonus (+7%) because back-action has not yet engaged while redistributing part of the peak into a two-line output and a broadened shoulder. (Relative to the *μ* = 0 symmetric platform’s own 205 μW peak, the design point delivers 47% at this drive: the price of the pump is paid partly in mistuning, partly in syphoning.) The second effect is the lever: it creates an entirely new harvesting region around 43 Hz, where the symmetric linear device delivers 0.15 μW. The net swept integral over 14–58 Hz is 448 μW⋅Hz against 354 for the linear baseline a factor 1.27, modest and honestly bounded by the load–pump conflict.

The conflict itself is quantitative and, once quantified, exploitable. Writing both loads as *R* = *ρR*_matched_, the effective dampings, the threshold *A*_thr_(*ρ*), and the coupled-branch line power all become explicit functions of *ρ*; Figure 8b maps them. Lightening the load reopens the pump *A*_thr_ falls monotonically toward its unloaded value at the price of per-amplitude conversion efficiency, and the product has an interior optimum: at 0.05 g, the coupled-branch power at the band centre peaks at *ρ*^⋆^ = 0.34. The full model validates the corrected theory to 1.5% at this point (20.1 μW simulated vs. 20.4 predicted, with the subharmonic demonstrably locked, *a*_1_ = 6 × 10^−6^), and a simulation at *ρ* = 0.5 lands on the activation boundary, falling back to the linear branch at comparable power; the trade-off is soft, which is itself useful. At higher ambient levels, the coupled branch pulls away (the 0.10 g curve), so the correct operating doctrine is load scheduling: matched load for broadband linear operation, light load (*ρ* ≈ *ρ*^⋆^) to engage the pump when the source concentrates near ${\tilde{f}}_{2}$.

Figure 8c states the application case plainly, for a line source at 43.3 Hz and 0.05 g an excitation representative of the rotating-machinery and compressor lines in the 40–50 Hz band tabulated in ambient-vibration surveys [4]. Within the fixed platform, the symmetric and mistuned linear devices deliver 0.15 and 0.21 μW; the slit-element device delivers 16.6 μW at matched load and 20.1 μW coupled at *ρ*^⋆^. The honest scope of that comparison is multi-line service from one monolithic device: a dedicated plain cantilever of the same laminate, length-tuned to the line (*L* = 124.1 mm), delivers 58.7 μW there about three times the element device but serves only that line, whereas the slit-element device simultaneously holds its ${\tilde{f}}_{1}$ line at the 96–154 μW level. The internal resonance contributes a further 21% at this level, together with the flat-topped band, the subharmonic second line, and the saturation cap that protects the magnet gap. The division of labour is the honest conclusion: at ambient levels, the lever earns the multi-line capability, the pump earns robustness and grows in value with drive.

### 4.8. Case B In Silico: Single-Tone Second-Harmonic Generation

**Figure 9:**
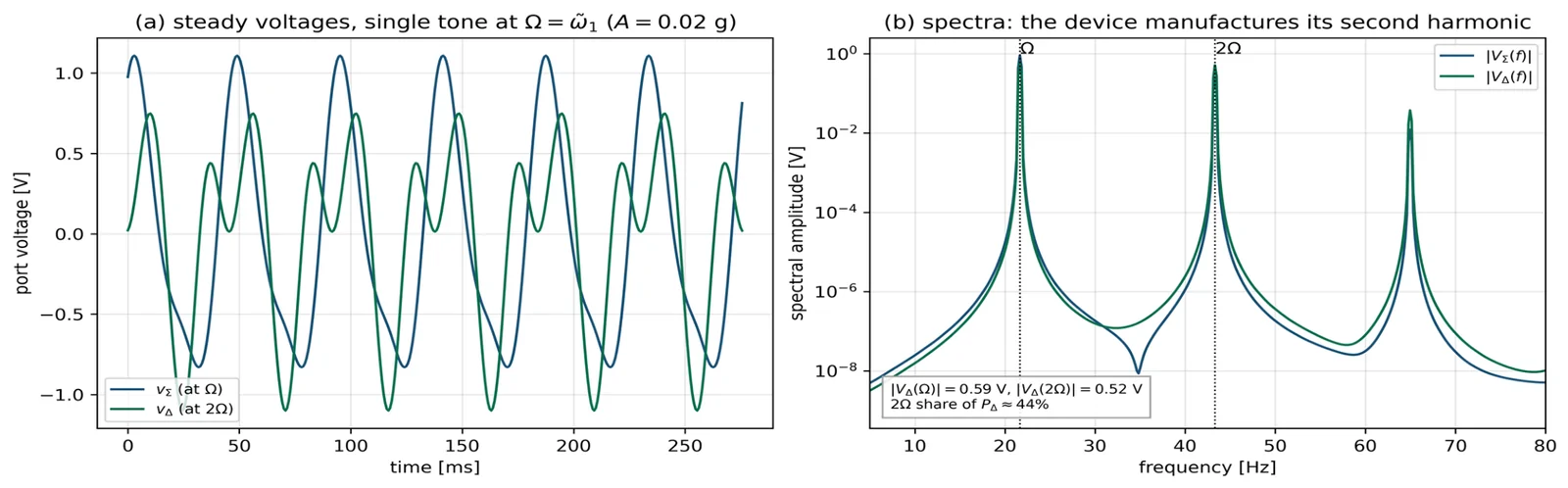
Case B with the full electromechanical model: Single tone at $\Omega ={\tilde{\omega }}_{1}$, *A* = 0.02 g. (**a**) Steady port voltages: *v*_Σ_ at *Ω*; *v*_Δ_ carries both lines, |*V*_Δ_(*Ω*)| = 0.59 V and |*V*_Δ_(2*Ω*)| = 0.52 V. (**b**) Spectra: The device manufactures its own second harmonic; the ports deliver equal power (11.7 μW each), with the 2*Ω* line carrying ≈ 44% of *P*_Δ_, i.e., ≈22% of the total output.

Figure 9 demonstrates the thresholdless regime with the full electromechanical model. A single tone at $\Omega ={\tilde{\omega }}_{1}$ and 0.02 g produces, in steady state, the fundamental on the sum port and a strong second-harmonic line on the differential port |*V*_Δ_(2*Ω*)| = 0.52 V against |*V*_Δ_(*Ω*)| = 0.59 V, so the second harmonic carries ≈44% of *P*_Δ_ (about 22% of the total output) with the power split equally between the ports: *P*_Σ_ = *P*_Δ_ = 11.7 μW. The differential excursion stays at 4% of the magnet gap, deep inside the validity of the force expansion. The physics is resonant frequency doubling by design: the quadratic slit force rectifies the in-phase motion into a differential drive at 2*Ω*, which lands exactly on ${\tilde{\omega }}_{2}$ because the element itself was used to enforce ${\tilde{\omega }}_{2}=2{\tilde{\omega }}_{1}$; the transfer follows the self-consistent version of Equation (16), in which the back-action appears as nonlinear damping and detuning on the driven mode:

(19) $$\left[{\left({\tilde{m}}_{1}+Q\;{\tilde{m}}_{2}\;a_{1}^{2}\right)}^{2}+{\left(\sigma_{1}-Q\;\sigma_{2c}\;a_{1}^{2}\right)}^{2}\right]a_{1}^{2}=\frac{{\tilde{\Gamma }}_{1}^{2}A^{2}}{4{\tilde{\omega }}_{1}^{2}},\;\;Q=\frac{\Lambda^{2}}{8{\tilde{\omega }}_{1}{\tilde{\omega }}_{2}\left({\tilde{m}}_{2}^{2}+\sigma_{2c}^{2}\right)},$$

with ${\tilde{m}}_{r}=\zeta_{r}{\tilde{\omega }}_{r}$, *σ*_1_ the drive detuning, $\sigma_{2c}=2\Omega -{\tilde{\omega }}_{2}$, and $a_{2}=\Lambda a_{1}^{2}/\left[4{\tilde{\omega }}_{2}{\left({\tilde{m}}_{2}^{2}+\sigma_{2c}^{2}\right)}^{1/2}\right]$; the cubic in $a_{1}^{2}$ bounds the response without any activation threshold, and the quantitative curves reported here are obtained by direct integration of the full electromechanical model. Beyond harvesting, a monolithic beam that routes half of a single-tone input’s electrical output through a separate differential port with a phase-locked second harmonic carrying ≈44% of that port’s power is a passive frequency-conversion element in its own right.

## 5. Design Guidelines

Table 4 consolidates the design point so that every quantitative claim is reproducible without archaeology; the findings then compress into a placement-first workflow.

The linear skeleton is set first. Fix *f*_1_ by slit length, width and total tip mass exactly as in the linear methodology of [31,32]; at *μ* = 0, the element provably cannot move it, while at the mistuned 2:1 design point, the residual shift is finite and must be budgeted (5.9% at *μ* = 0.8 here; Section 4.7). Then place *f*_2_ with the element polarity and strength via Equation (9), staying above $k_{1}^{\mathrm{cr}}$ unless differential bistability is the goal.

For 2:1 operation with the repulsive polarity (the attractive polarity opens the complementary window *r* < 2 from above; the computed 117 mm example is in Section 3.2), mistuning must first open the window from below: the bare ratio *f*_2_/*f*_1_ rises with *μ* and crosses 2 at *μ*^⋆^ = 0.554 for the present geometry (Appendix A, Figure A1b), so the lever can pull ${\tilde{f}}_{2}$ down onto $2{\tilde{f}}_{1}$ only for *μ* > *μ*^⋆^; the validity rule 𝒫 ≤ 0.1 then sets the working floor *μ* ≥ 0.72, and fabrication of the light prong mass bounds the top, giving the recommended window 0.72 ≲ *μ* ≲ 0.85 (Figure A1c). Mounting at $\Delta_{0}^{⋆}≃0.246\;g_{0}$ then kills the cubic.

The electrical side follows. Wire the sum port for the primary/subharmonic power and the differential port for the saturated/second-harmonic output; the anti-resonance switching of the linear device carries over per port. Treat *A*_thr_ from Equation (15) as the commissioning number, since measured activation at the predicted threshold is the sharpest field test of the whole model. Finally, schedule the load: matched resistances suppress the pump (the loaded threshold is 4.6× the mechanical one here); to engage the internal resonance at ambient levels, lighten both ports to *ρ*^⋆^ ≈ 0.3–0.4 of matched (Figure 8b), and switch back to matched for broadband linear operation.

## 6. Limitations and Future Work

Seven limitations are declared. Reconstructed geometry: The working geometry reproduces the published 117 mm anchors exactly and the 10 g case within 2–4%, but misses the 90 mm symmetric-mass *f*_2_ by −15%; all quantitative design-point values await recalibration to the fabricated device, while the structural theorems are geometry-independent. Element model: The point-dipole law Equation (3) is a far-field idealisation; finite-magnet corrections shift *k_n_* and $\Delta_{0}^{⋆}$, though the existence of the $U_{s}^{\left(4\right)}$ zero is generic. Damping and loading: Modal damping on the shifted modes is an assumption, and all mechanical thresholds are load-dependent; the matched two-port load raises *A*_thr_ by 4.6× here (Section 4.7), so quoted thresholds must always name their electrical condition; the demonstrated sensitivity (a 20% frequency shift mis-scales the response by the same fraction if damping is assigned to the wrong basis) makes experimental damping identification on the assembled device essential. Eddy-current damping*:* NdFeB magnets moving millimetres from aluminium prongs dissipate in the differential coordinate whose damping sets *A*_thr_ linearly; an image-dipole estimate for the reference pair (*m* ≈ 0.028 A m ^2^, standoff 3.5 mm, prong thickness 0.72 mm) gives *c_e_* ∼ 1.3 × 10^−3^ N s/m, i.e., *ζ*_2,add_ ≈ 8 × 10^−4^ about 7% of the assumed *ζ_m_*, raising *A*_thr_ by the same fraction (with an 𝒪(1) geometric factor; slotting the facing prong surface or increasing standoff suppresses it). Magnet mass and rotation: the quoted tip masses 10.8/1.2 g are total tip inertias, including magnet and shim (a ∼3 mm N42 cube is ≈0.2 g, i.e., 17% of the lighter mass not negligible, hence folded in); tip rotation is bounded directly from the eigenfunctions, |*φ*_2*a*_′(*L*)| = 85.8 giving 0.9 mrad at the largest reported response and a pole-centre shift of 1.4 μm 0.11% of $\Delta_{0}^{⋆}$ so rotation-induced changes of $k_{2},k_{3},\Delta_{0}^{⋆}$ are sub-percent at operating amplitudes. The model carries no torsional degree of freedom, so any twisting the magnets’ off-centroid placement might excite (Figure 1a) is not represented in Equation (4) directly; the independent plate cross-check of Section 3.4, which does carry full twist energy, closely reproduces the beam model regardless, with its one substantial discrepancy (*f*_2_, Section 3.4) independently attributed there to chordwise compliance, not torsion. Beam-intrinsic nonlinearity: Proposition 1 removes the element from the symmetric family; it does not make that family linear. Geometric nonlinearity detunes the first mode by its half-bandwidth only at tip amplitudes $a^{⋆}∼L\sqrt{\zeta_{1}/\kappa }\approx 8$ mm (*κ* = 𝒪(3)), a factor of more than 20 above the largest amplitude reported here (0.35 mm), so the exactly linear-symmetric-family claim is scoped to *a*_1_≪*a*^⋆^; on the coupled branch, where *a*_1_ grows with drive, this is the amplitude at which the quadratic closure hands over to beam physics. No experiment yet: every claim here is theoretical–computational, with internal verification only. The natural experimental programme is staged: (i) linear lever and *f*_1_-invariance test with a micrometre-gapped magnet pair; (ii) static pitchfork and hysteresis under gap sweep; (iii) threshold–saturation curve at the 2:1 point, including the *A*_thr_ ∝ *μ*^−3^ law across a mass-mistuning series; (iv) two-port power characterisation against the linear baseline. Harmonic balance with continuation, and stochastic (band-limited) excitation studies in the spirit of [8,20], are the immediate modelling extensions, while the shell finite-element cross-check of Section 3.4 should be extended to the fabricated three-dimensional geometry with the measured mass blocks.

## 7. Conclusions

A compact magnetic element mounted inside the slit of a monolithic slitted piezoelectric harvester acts on the prong-difference coordinate alone, and symmetry then does work that tuning cannot. At equal tip masses, the element is invisible, at every order in amplitude, to the symmetric mode family, so the base-excited resonance stays exactly linear and pinned; a rank-one lever places the anti-symmetric resonance across 33–60 Hz with the sign of the magnet polarity; a supercritical pitchfork provides bistability confined to the differential coordinate; and deliberate mass mistuning opens a gated 2:1 internal resonance whose coupling grows asymptotically as *μ*^2^ and whose activation threshold falls as *μ*^−3^.

At the design point (*μ* = 0.8, ${\tilde{f}}_{1,2}=21.66/43.32$ Hz), the channel activates at 0.017 g unloaded and 0.079 g under matched loads, saturates the driven mode independently of forcing, spills a subharmonic onto the power-carrying mode, and generates the second harmonic on the differential port from a single tone, with the 2*Ω* line carrying about 44% of that port’s power. The gains are quantified against their cost: the first resonance shifts by 5.9% at the mistuned design point, and the threshold-free transfer syphons 38% of the matched-load power at 0.05 g; in return, the device serves a 43.3 Hz line at 16.6–20.1 μW, where the symmetric platform delivers 0.15–0.21 μW and integrates 448 against 354 μW·Hz over the band (1.27×). Load and pump compete matched resistances raise the activation threshold 4.6×, and engaging the internal resonance at ambient levels requires lightening both ports to ρ^*^ ≈ 0.3 − 0.4 of matched.

The modulation theory and direct integration of the full cubic model agree to 0.1–2% at the tuned drive, inside a validity window bounded by two explicit rules: the parasitic–quadratic ratio 𝒫 ≤ 0.1, which sets the mistuning floor *μ* ≥ 0.72, and mounting at the offset $\Delta_{0}^{⋆}$ that removes the cubic. An independent finite-element model confirms that the selection rules and participations are robust to the model family, while the anti-symmetric frequency itself is model-form sensitive; and the full model shows the first-order activation tongue to be an upper bound off-centre, with the fold and its hysteresis realised on the negative-detuning flank (activation ≈ 0.07 g, fold ≈ 0.045 g at *σ*_2_ = −8 rad/s) and lost on the positive one. These limits are stated rather than hidden, and they mark where a second-order closure and, above all, experiment must take over.

The broader claim is architectural. In slitted and split multimodal harvesters, symmetry-selective placement of nonlinearity lets linear reliability and nonlinear functionality coexist by construction, turning the coupling between modes from an accident of geometry into a designed, gated, and quantitatively predictable channel. The placement-first workflow of Section 5 reduces this to a short sequence of decisions, and the predicted activation threshold is its sharpest field test.

## Figures and Tables

**Table 1:** Design map of the slit element. Each nonlinear regime is switched on by an explicit, independently controllable symmetry breaking. The *μ* = 0 invisibility (first row) and the mistuned 2:1 pump (third row) are mutually exclusive operating regimes: at the mistuned design point the residual *f*_1_ shift (5.9%) and the thresholdless syphoning are quantified in Section 4.7.

Δ_0_	*μ*	Tuning	Regime
any	0	any	element invisible to 𝒮 (exact at *μ* = 0); *f*_2_ lever
0	0	*C* > *C_cr_*	differential bistability (pitchfork in Δ)
≠0	≠0	*ω*_2_≃2*ω*_1_	2:1 autoparametric transfer, *Λ* ∝ *μ*^2^, *A*_thr_ ∝ *μ*^−3^
0	≠0	*ω*_2_≃3*ω*_1_	1:3 cubic interaction

**Table 2:** Verification battery. “Exact” denotes machine-precision identities; percentages are worst-case deviations.

Check	Quantity	Outcome
V1	no-slit uniform limit	41.8000 Hz, error 9 × 10^−7^ %
V2	anchors of Ref. [31]	fitted exactly; 10 g predicted to −4.4/+1.9 %;
		90 mm *f*_2_ misses by −15% (declared)
V3	selectivity and orthonormality	${\left|d_{r}\right|}_{S}\;∼\;10^{-14}$, |*Γ*|_𝒜_ ∼ 10^−16^, max|*G* − *I*| ∼ 4 × 10^−13^
V4	rank-one lever	Δ*f*_1_ = 0 exactly; *f*_2_ closed form to 22 mHz; $k_{1}^{\mathrm{cr}}$ to 0.2%
V5	force consistency	Jacobian symmetric; 2:1 coefficient ratio = 2.000000
V6	mistuning law Equation (8)	0.05% at *μ* = 0.05
V7	MMS vs. full model	0.1–2% across all branches

**Table 3:** Independent FE (Morley plate) vs. branched-beam model at the reconstructed geometry (slit 90 mm). *d_r_* and *Γ_r_* are mass-normalised participations; “≈0” denotes a magnitude below 10^−4^ relative.

Quantity	Beam	Plate FE	Deviation	Verdict
*f*_1_ (*μ* = 0) [Hz]	24.53	24.99	+1.9%	agree
*f*_2_ (*μ* = 0, anti-sym.) [Hz]	42.19	35.59	−15.6%	model-form sensitive
*d*_1_ (*μ* = 0)	0 (exact)	≈0		theorem holds
*Γ*_2_ (*μ* = 0)	0 (exact)	≈0		theorem holds
*d*_2_ (*μ* = 0)	15.72	15.47	−1.6%	agree
*d*_2_ (*μ* = 0.8)	18.59	17.87	−3.9%	agree
*d*_1_(0.4)/*d*_1_(0.8)	0.63	0.72		same gating shape

**Table 4:** Consolidated design-point parameters (slit 90 mm, *M*_1_/*M*_2_ = 10.8/1.2 g incl. magnets, *μ* = 0.8, gap *g*_0_ = 5 mm, $\Delta_{0}=\Delta_{0}^{⋆}=1.229$ mm). Modal quantities use the mass-normalised convention of Section 2.3: ${\tilde{d}}_{r}$ in kg^−1/2^, ${\tilde{\Gamma }}_{r}$ in kg^1/2^, $\tilde{\theta }$ in N V^−1^ kg^−1/2^, *Λ* in m^−1^ kg^−1/2^ s^−2^, $a_{2}^{\mathrm{sat}}$ in m kg^1/2^; the parenthetical gives the physical differential tip amplitude $\left|{\tilde{d}}_{2}\right|\;a_{2}^{\mathrm{sat}}$.

Quantity	Symbol	Value
Linear skeleton (μ = 0.8, slit 90 mm)		
Bare natural frequencies	*f*_1_,*f*_2_	23.02, 54.25 Hz
Diagonalized frequencies at the 2:1 design point	${\tilde{f}}_{1},{\tilde{f}}_{2}$	21.66, 43.32 Hz
Differential participations	${\tilde{d}}_{1},{\tilde{d}}_{2}$	−5.81, 17.96
Base-excitation participations	${\tilde{\Gamma }}_{1},{\tilde{\Gamma }}_{2}$	−0.146, −0.067
Slit element		
Linear stiffness	*k*_1_	−124.9 N/m
Critical (buckling) stiffness at μ = 0.8	$k_{1}^{\mathrm{cr}}$	−286.8 N/m
Quadratic stiffness	*k*_2_	1.155 × 10^5^ N/m^2^
Cubic stiffness	*k*_3_	0 (by construction, Equation (18))
Coupling and thresholds		
2:1 coupling coefficient	*Λ*	6.996 × 10^7^
Activation threshold, unloaded	*A*_thr_	0.169 m/s^2^ = 0.017 g
Activation threshold, matched load	$A_{\mathrm{thr}}^{\mathrm{loaded}}$	0.079 g
Saturation amplitude of mode 2	$a_{2}^{\mathrm{sat}}$	6.35 × 10^−6^ (0.11 mm tip)
Ports, loads and damping		
Sum-port couplings	${\tilde{\theta }}_{\Sigma }$	0.0108, 0.0121
Difference-port couplings	${\tilde{\theta }}_{\Delta }$	0.0076, −0.0230
Port capacitances	*C_p_*_Σ_,*C_p_*_Δ_	196.8, 138.3 nF
Matched load resistances	*R*_Σ_,*R*_Δ_	37.3, 26.6 k*Ω*
Mechanical damping ratio	*ζ_m_*	0.012
Electrical damping ratios, matched loads	*ζ_e_*_,1_,*ζ_e_*_,2_	0.0125, 0.0150

## Data Availability

The data and code supporting the findings of this study are available from the corresponding author upon reasonable request.

## Nomenclature

Latin symbols	
*a_r_*	Slow-flow amplitude of mode *r* (m kg^1/2^)
$a_{2}^{\mathrm{sat}}$	Saturation amplitude of mode 2 on the coupled branch (m kg^1/2^)
*A*	Base-acceleration amplitude (m s^−2^; quoted in g)
*A*_thr_	Activation threshold of the 2:1 auto-parametric transfer (m s^−2^; quoted in g)
*b*	Beam width (m)
*b_p_*	Prong width, (*b* − *w_s_*)/2 (m)
*C*	Dipole interaction strength in the element law *U_s_*(*s*) (J m^3^)
*C_p_*_Σ_, *C_p_*_Δ_	Sum-/difference-port piezoelectric capacitances (F)
*c_e_*	Eddy-current drag coefficient (limitations estimate) (N s m^−1^)
*D*	Plate flexural rigidity (finite-element cross-check) (N m)
*d_r_*, ${\tilde{d}}_{r}$	Differential tip participation of mode *r* (bare/diagonalised) (kg^−1/2^)
$\hat{\mathrm{EI}}$	Composite flexural rigidity per unit width (N m)
*E_s_*, *E_p_*	Young’s moduli of substrate and piezoelectric layers (Pa)
*f_r_*, ${\tilde{f}}_{r}$	Natural frequencies of mode *r* (bare/diagonalised) (Hz)
*g*_0_	Magnet gap across the slit (m)
*h_s_*, *h_p_*	Substrate and piezoelectric layer thicknesses (m)
*k*_1_, *k*_2_, *k*_3_	Linear, quadratic, cubic element stiffness coefficients (N m^−1^, N m^−2^, N m^−3^)
$k_{1}^{\mathrm{cr}}$	Critical linear stiffness of the differential pitchfork (N m^−1^)
*L*, *L*_1_, *L_s_*	Beam length; root (un-slit) length; slit length (m)
*M*_1_, *M*_2_, $\bar{M}$	Tip masses (totals incl. magnet and shim); their mean (kg)
$\hat{m}$	Areal mass density of the laminate (kg m^−2^)
*m*	Magnet dipole moment (A m^2^)
${\tilde{m}}_{r}$	Slow-flow damping parameter, $\zeta_{r}{\tilde{\omega }}_{r}$ (s^−1^)
𝒫	Parasitic quadratic ratio (validity rule 𝒫 ≤ 0.1)
*P*	Harvested electrical power (W)
*Q*	Case-B back-action coefficient (self-consistent closure) (m^−2^ kg^−1^ s^−1^)
*q_r_*	Mass-normalised modal coordinate of mode *r* (m kg^1/2^)
*r*	Bare frequency ratio, *f*_2_/*f*_1_
*R*_Σ_, *R*_Δ_	Sum-/difference-port load resistances (*Ω*)
*s*	Relative transverse offset of the magnet pair (m)
**T**	Rotation diagonalizing the *k*_1_-shifted linear part
*U_s_*	Transverse dipole interaction potential of the element (J)
*v*_Σ_, *v*_Δ_	Sum- and difference-port voltages (V)
*w_a_*, *w_b_*	Deflections of prongs a and b (m)
*w_s_*	Slit width (m)
Greek symbols	
*Γ_r_*, ${\tilde{\Gamma }}_{r}$	Base-excitation participation of mode *r* (kg^1/2^)
*γ*_1_, *γ*_2_	Autonomous slow phases of the modulation equations (rad)
Δ	Differential coordinate, *w_a_* − *w_b_* (m)
Δ_0_, $\Delta_{0}^{⋆}$	Static mounting offset; offset at which $U_{s}^{\left(4\right)}=0$ (m)
*ζ_m_*, *ζ_e_*_,*r*_, *ζ_r_*	Mechanical, electrical (load), and total damping ratios
*θ*_Σ_, *θ*_Δ_ (and $\tilde{\theta }$)	Port electromechanical couplings per mode (N V^−1^ kg^−1/2^)
*κ*	Geometric-nonlinearity coefficient of the bare beam
*Λ*	Quadratic 2:1 coupling coefficient, $k_{2}{\tilde{d}}_{1}^{2}{\tilde{d}}_{2}$ (m^−1^ kg^−1/2^ s^−2^)
*λ*	Frequency-scale factor in the uncertainty bracket
*μ*	Mass mistuning, (*M*_1_ − *M*_2_)/(*M*_1_ + *M*_2_)
*μ* ^*^	Repulsive-lever floor (bare *r* = 2 crossing)
*ν*	Poisson’s ratio (plate cross-check)
*ρ*, *ρ*^*^	Load scale relative to matched; its coupled-branch optimum
*ρ_s_*, *ρ_p_*	Densities of substrate and piezoelectric layers (kg m^−3^)
*σ*_1_, *σ*_2_, *σ*_2*c*_	Internal, external, and Case-B detuning parameters (rad s^−1^)
*φ_r_*	Mass-normalised mode shape of mode *r* (kg^−1/2^)
*χ*	Mistuning gating coefficient, *d*_1_≃*χμd*_2_
*Ω*	Drive (base-excitation) angular frequency (rad s^−1^)
*ω_r_*, ${\tilde{\omega }}_{r}$	Angular natural frequencies (bare/diagonalized) (rad s^−1^)
Subscripts, superscripts, and accents	
$\left(\;\tilde{\cdot }\;\right)$	Evaluated in the basis diagonalizing the *k*_1_-shifted linear part
$\left(\;\hat{\cdot }\;\right)$	Composite (per-unit-width or areal) laminate property
(⋅)*_a_*, (⋅)*_b_*	Prong a (heavy side) and prong b (light side)
(⋅)_Σ_, (⋅)_Δ_	Sum (in-phase) and difference (differential) electrical ports
(⋅)*_r_*	Mode index, *r* = 1,2,…
𝒮, 𝒜	Symmetric and anti-symmetric mode families
(⋅)^⋆^, (⋅)^*^	Distinguished value (zero of $U_{s}^{\left(4\right)}$; optimum; floor)
(⋅)^(4)^	Fourth derivative with respect to the argument
(⋅)^cr^, (⋅)^sat^, (⋅)_thr_	Critical, saturation, and threshold values
Abbreviations	
2-DOF	Two degrees of freedom
FE	Finite element
MDPI/ACS	Reference-list style of the target journal
MMS	Method of multiple scales
ODE	Ordinary differential equation (direct integration of the full model)
PZT	Lead zirconate titanate (piezoelectric ceramic)

## Appendix A. Limitations of the Passive Linear Device, Re-Derived

This appendix makes the present paper self-contained with respect to the two limitations of the linear slitted device that motivate the slit element, using only Section 2 machinery, and derives the mistuning window quoted in the design guidelines.

### Appendix A.1. The Anti-Symmetric Family Cannot Be Powered at Symmetric Masses

At *μ* = 0, the geometry and mass distribution are invariant under the mirror *y* → −*y*; every mode is either symmetric or anti-symmetric, and for anti-symmetric modes the base-participation integral $\Gamma_{r}=\int \hat{m}\;\varphi_{r}\;dx+\sum_{k}M_{k}\varphi_{r}\left(x_{k}\right)$ vanishes identically because the integrand is odd. Base excitation therefore delivers zero linear drive to the entire anti-symmetric family: the second power peak of a symmetric-mass slitted device is fed only by fabrication asymmetries, which is why it was observed at the parasitic ∼15% level in the fabricated device [31] and why no passive symmetric design can raise it. Powering *f*_2_ linearly requires *μ* > 0, which entangles placement, as follows.

### Appendix A.2. The Placement Locus

Figure A1a maps the bare ratio *r* = *f*_2_/*f*_1_ over slit length and symmetric tip mass for the calibrated laminate: *r* spans 1.1–6.7 and is governed almost entirely by slit length, while *f*_1_ (18.8–33.1 Hz over 2–12 g) is governed almost entirely by mass. Frequencies alone are thus two-knob placeable but by Appendix A.1 the anti-symmetric mode so placed carries no base power and no coupling, and the third target (powering *f*_2_) is unreachable for the passive device. The declared calibration limit is visible on the same panel: the model point at (90 mm,6 g) sits at *r* = 1.72 against the published anchor 2.10, the −15% anti-symmetric miss of Section 3.2, consistent with the strong model-form sensitivity of that branch demonstrated by the finite-element cross-check of Section 3.4 (which itself sits below the beam the direction question is discussed there).

### Appendix A.3. The Mistuning Path and the 2:1 Window

Powering the second peak with *μ* > 0 moves the pair along a fixed curve: Figure A1b shows *r*(*μ*) at the 90 mm geometry rising from 1.72 to 2.71, crossing *r* = 2 at *μ*^⋆^ = 0.554. Since the repulsive element polarity (*k*_1_ < 0) can only lower ${\tilde{f}}_{2}$, the 2:1 condition ${\tilde{f}}_{2}=2{\tilde{f}}_{1}$ is reachable only for *μ* > *μ*^⋆^ a floor, not a ceiling. At the exactly anchored 117 mm geometry the curve sits lower still, *r*(0.8) = 1.86 < 2, which is why that geometry cannot host the repulsive-polarity 2:1 design; the attractive-polarity design there is viable and quantified in Section 3.2, and the 90 mm class is retained for its 3.4× lower activation threshold. The parasitic-quadratic validity rule 𝒫(*μ*) ≤ 0.1 then sets the working floor *μ* ≥ 0.72 (Figure A1c), and fabricability of the light prong mass (*M*_2_ ≳ 0.9 g at $\bar{M}=6$ g) bounds the top, giving the recommended window 0.72 ≲ *μ* ≲ 0.85 quoted in the design guidelines. The lever itself also bounds the top of the window: the lowest ratio ${\tilde{f}}_{2}/{\tilde{f}}_{1}$ it can reach rises with *μ* (Figure A2a) and crosses 2 at *μ* = 0.85, the same value as the fabricability bound, so the recommended window is unchanged; with the anti-symmetric branch 17.6% stiffer than modelled that crossing moves down to *μ* = 0.66, below the validity floor the sensitivity quantified in Section 3.2.

## Appendix B. Multiple-Scales Derivation of the Slow Flows

The reduced two-mode system in the shifted basis is ${\ddot{q}}_{1}+2\zeta_{1}{\tilde{\omega }}_{1}{\dot{q}}_{1}+{\tilde{\omega }}_{1}^{2}q_{1}+2\Lambda q_{1}q_{2}={\tilde{\Gamma }}_{1}A\cos\mathrm{\Omega t}$, ${\ddot{q}}_{2}+2\zeta_{2}{\tilde{\omega }}_{2}{\dot{q}}_{2}+{\tilde{\omega }}_{2}^{2}q_{2}+\Lambda q_{1}^{2}={\tilde{\Gamma }}_{2}A\cos\mathrm{\Omega t}$, with the (2*Λ*,*Λ*) coefficient pair descending from the single potential term $\Lambda q_{1}^{2}q_{2}$. Introduce *ε* ordering with damping, forcing and quadratic coupling at first order; write $q_{r}=a_{r}\left(T_{1}\right)\cos\left[{\tilde{\omega }}_{r}T_{0}+\beta_{r}\left(T_{1}\right)\right]$ with *T_n_* = *ε^n^t*, and impose the internal detuning ${\tilde{\omega }}_{2}=2{\tilde{\omega }}_{1}+\varepsilon \sigma_{1}$. Eliminating secular terms at 𝒪(*ε*) gives, for Case A ($\Omega ={\tilde{\omega }}_{2}+\varepsilon \sigma_{2}$), the autonomous phases *γ*_1_ = *β*_2_ − 2*β*_1_ + *σ*_1_*T*_1_ and *γ*_2_ = *σ*_2_*T*_1_ − *β*_2_, and the slow flow quoted in Section 2.7; setting *a*_1_′ = *a*_2_′ = *γ*_1_′ = *γ*_2_′ = 0 and eliminating the phases by squaring yields the saturation amplitude Equation (14) on the coupled branch and, from the marginal condition on the trivial branch, the activation threshold Equation (15). For Case B ($\Omega ={\tilde{\omega }}_{1}+\varepsilon \sigma_{2}$) only *q*_2_ is parametrically driven by $a_{1}^{2}$; its stationary amplitude is Equation (16), and retaining the back-action of *q*_2_ on *q*_1_ in the *q*_1_ secular condition gives the self-consistent closure Equation (19) of Section 4.7. The entire derivation was additionally re-executed symbolically (computer algebra) as verification stage V5 of Table 2.
